# Integrated analysis of transcriptome and small RNAome reveals regulatory network of rapid and long-term response to heat stress in *Rhododendron moulmainense*

**DOI:** 10.1007/s00425-024-04375-5

**Published:** 2024-03-29

**Authors:** Si-Jia Liu, Chang Cai, Hong-Yue Cai, Yu-Qing Bai, Ding-Yue Wang, Hua Zhang, Jin-Gen Peng, Li-Juan Xie

**Affiliations:** 1 College of Architectural Engineering, Shenzhen Polytechnic University, Shenzhen, 518055 China; 2 Guizhou Provincial Key Laboratory for Information Systems of Mountainous Areas and Protection of Ecological Environment, Guizhou Normal University, Guiyang, 550001 China; 3 Administrative Office of Wutong Mountain National Park, Shenzhen, 518004 China

**Keywords:** Heat stress, Long-term adaptability, Multiomics analysis, Rapid response, Regulatory network, *Rhododendron moulmainense*

## Abstract

**Main conclusion:**

**The post-transcriptional gene regulatory pathway and small RNA pathway play important roles in regulating the rapid and long-term response of Rhododendron moulmainense to high-temperature stress.**

**Abstract:**

The Rhododendron plays an important role in maintaining ecological balance. However, it is difficult to domesticate for use in urban ecosystems due to their strict optimum growth temperature condition, and its evolution and adaptation are little known. Here, we combined transcriptome and small RNAome to reveal the rapid response and long-term adaptability regulation strategies in *Rhododendron moulmainense* under high-temperature stress. The post-transcriptional gene regulatory pathway plays important roles in stress response, in which the protein folding pathway is rapidly induced at 4 h after heat stress, and alternative splicing plays an important role in regulating gene expression at 7 days after heat stress. The chloroplasts oxidative damage is the main factor inhibiting photosynthesis efficiency. Through WGCNA analysis, we identified gene association patterns and potential key regulatory genes responsible for maintaining the ROS steady-state under heat stress. Finally, we found that the sRNA synthesis pathway is induced under heat stress. Combined with small RNAome, we found that more miRNAs are significantly changed under long-term heat stress. Furthermore, MYBs might play a central role in target gene interaction network of differentially expressed miRNAs in *R. moulmainense* under heat stress. MYBs are closely related to ABA, consistently, ABA synthesis and signaling pathways are significantly inhibited, and the change in stomatal aperture is not obvious under heat stress. Taken together, we gained valuable insights into the transplantation and long-term conservation domestication of *Rhododendron*, and provide genetic resources for genetic modification and molecular breeding to improve heat resistance in *Rhododendron*.

**Supplementary Information:**

The online version contains supplementary material available at 10.1007/s00425-024-04375-5.

## Introduction

*Rhododendron moulmainense* (*R. moulmainense*), a member of the family Ericaceae and subgenus *Azaleastrum*, is native to China and known as the world's only large tree azalea found in the southernmost and lowest regions (Kang et al. 2009). Flourishing at elevations of around 600–1500 m, this evergreen species boasts splendid tree shapes and vibrant flowers, creating a unique and visually captivating plant landscape. In addition, it is also useful in food and medicine (Jing and Wang 2018). However, when transplanted to lower altitudes, *R. moulmainense* experiences leaf yellowing, burning, and even mortality. The molecular mechanisms underlying the adaptation of *R. moulmainense* to low-elevation environments remain elusive. Previous studies have explored the impacts of light intensity, soil drainage, and heat stress on *R. moulmainense* growth (Wang et al. 2011; Hong et al. 2016; Bai et al. 2017), but the regulatory pathways responsible for its responses to these environmental factors remain unclear. Therefore, conducting whole-genome analysis of *R. moulmainense*'s heat stress rapid responses and long-term adaption will aid in identifying key regulators and pathways that could be targeted for enhancing thermotolerance. Understanding the regulatory mechanisms of *R. moulmainense* in low-altitude adaptation will broaden our comprehension of high-altitude azaleas and promote their domestication and application.

Heat stress poses a significant limitation in the process of introducing and domesticating *Rhododendron* at low altitudes (Wang et al. 2021a). At the cellular level, heat stress affects critical plant growth and developmental processes by altering membrane fluidity and disrupting protein homeostasis (Bokszczanin et al. 2013). Plants have evolved various thermotolerance strategies to cope with unusually high temperatures, involving factors such as small RNAs (sRNAs), heat shock transcription factors (HSFs), heat shock proteins (HSPs), and reactive oxygen species (ROS)-scavenging enzymes (Ohama et al. 2017; Zhao et al. 2020). However, there have been limited reports on the high-temperature responding factors of high-altitude rhododendron (Wang et al. 2021a). Considering that the subgenus *Azaleastrum* serves as an evolutionary link between *Rhododendrons* and *Azaleas* (Ming and Fang 1990), and it is believed to be an early subgenus that diverged from the *Rhododendron* ancestor based on molecular phylogenetic studies using chloroplast genes and nuclear regions (Shrestha et al. 2018; Wang et al. 2021a), the subgenus *Azaleastrum* plays a crucial role in evolution and adaptation within the genus *Rhododendron*. Therefore, understanding the heat stress regulatory network in *R. moulmainense* will provide genetic resources for genetic modification and molecular breeding of Rhododendron in low-altitude environments adaption.

Small RNAs (sRNAs) are non-coding RNAs consisting of 20–24 nucleotides that modulate gene expression by causing transcriptional gene silencing or post-transcriptional gene silencing (Axtell 2013; D'Ario et al. 2017; Yu et al. 2019). They are responsible for plant thermotolerance and heat stress responses by modulating various molecular pathways (Ruiz-Ferrer and Voinnet 2009; Khraiwesh et al. 2012; Shriram et al. 2016; Liu et al. 2017). Among sRNAs, microRNAs (miRNAs) constitute a subclass of endogenous sRNAs that primarily target mRNAs for degradation or translational repression (Chen and Rechavi 2022). MiRNAs play key roles in numerous physiological and developmental processes in plants, as well as in responses to environmental stresses (Ruiz-Ferrer and Voinnet 2009; Voinnet 2009; Rodriguez et al. 2016; Shriram et al. 2016). They can target genes that encode a wide variety of regulatory proteins, including a significant number of transcription factors (TFs), signifying the central role of miRNAs in gene regulatory networks. Typically, a single miRNA family could target several different target genes and participates in various aspects of stress tolerance and plant development. Growing evidence indicates that miRNAs play critical roles in orchestrating plant development and heat stress adaptation. For instance, miR156 can be highly induced under heat stress, promoting the continuous expression of heat stress response genes and enhancing plant thermotolerance (Stief et al. 2014). The miR172-*AP2* module also plays a significant role in plant heat stress response (Kouhi et al. 2020; Peng et al. 2020). As miRNAs are ubiquitous regulators in plant development, studying the miRNAs responsive to high-temperature stress in *R. moulmainense* and their specific downstream target genes will facilitate the introduction, domestication, and improvement of *Rhododendron* plants.

To address the gaps in our understanding of *Rhododendron* plants' thermotolerance under low-altitude high-temperature adaptation, we analyzed the heat stress regulatory network of *R. moulmainense*, a species within the subgenus *Azaleastrum* with a crucial evolutionary position in *Rhododendron*. *R. moulmainense* was subjected to 42 °C heat stress for 7 days (long term) or 4 h (short term), and the leaf tissues were harvested for mRNA sequencing (mRNA-seq) and sRNA sequencing (sRNA-seq). Through comparison of mRNA transcriptome profiles, we aimed to identify key genes regulating the expression of heat stress-related genes, with a notable role of endoplasmic reticulum (ER)-mediated protein folding in heat rapid response and spliceosome in heat long-term adaption. We also analyzed critical genes responsible for maintaining ROS steady state. Based on the sRNA transcriptomic analysis, we identified miRNAs that could regulate heat stress responses, and MYBs might play crucial role in regulating heat stress. This comprehensive expression profiling of heat-responsive sRNAs and mRNAs sheds light on the molecular mechanisms governing heat stress in *R. moulmainense* at both transcriptional and post-transcriptional levels. Unraveling the molecular mechanisms underlying *R. moulmainense*'s thermotolerance, particularly the pivotal regulatory factors, paves the way for genetic breeding of heat-resistant *R. moulmainense*.

## Materials and methods

### Plant materials and growth conditions

*R. moulmainense* plants were cultivated in the Nursery of Wutong Mountain Scenic Area for 3 years. Plants were exposed to a heat treatment of 42 °C, with consistent humidity, for durations of 4 h and 7 days. Following the heat treatment, leaves were collected and promptly frozen in liquid nitrogen. Each biological replicate was obtained by pooling samples from 3 individual plants, and a total of 3 biological replicates were performed.

### Weighted gene co-expression network analysis (WGCNA) of *R. moulmainense* transcriptome

The WGCNA package (https://horvath.genetics.ucla.edu/html/CoexpressionNetwork/Rpackages/WGCNA/) was employed to generate co-expression networks (Langfelder and Horvath 2008). The *K*-means clustering analysis was performed on the total differentially expressed genes (DEGs) from mock versus heat samples. Then, WGCNA was performed on the genes with reads per kilobase per million mapped reads (RPKMs). The automatic network construction function, blockwiseModules, was utilized to obtain the modules. The parameters used were networkType as 'signed', soft power as 9, minModuleSize as 30, minKMEtoStay as 0.3, and mergeCutHeight as 0.25. Out of the total 2903 genes, six trait-specific modules were identified, each consisting of closely related genes. The remaining 458 genes were considered outliers and were placed in the gray module. A list of these genes is provided in Suppl. Table S1.

### Measurement of chlorophyll fluorescence

Chlorophyll fluorescence was assessed in vivo using fully expanded leaves of *R. moulmainense*. The LI-6400XT photosynthesis system from Li-Cor Biosciences (Lincoln, Nebraska, USA) equipped with a leaf chamber fluorometer (Li-Cor Part No.6400-40, enclosed leaf area: 2 cm^2^) was utilized, following the manufacturer's instructions. The measurements were taken at a leaf temperature of ~ 22 °C, with the light source consisting of a mixture of blue (10%) and red (90%) LEDs.

### Analysis of H_2_O_2_ accumulation and malondialdehyde (MDA) accumulation

H_2_O_2_ accumulations in the assessed leaves of *R. moulmainense* were analyzed as per the provided guidelines by Solarbio (BC3950, Beijing, China). The levels of MDA in the examined leaves of *R. moulmainense* were measured following the instructions given by Solarbio (BC0020). The samples were collected from leaves of *R. moulmainense* treated with mock or heat at 4 h after heat treatment (HAH) or 7 days after heat treatment (DAH). Each biological replicate comprised of pooled samples from 3 individual plants, and a total of 3 biological replicates were conducted.

### Enzyme activity determination of catalase (CAT), superoxide dismutase (SOD), and ascorbate peroxidase (APX)

The enzyme activities of CAT, APX, and SOD were measured using the corresponding assay kits (Colorimetric) following the manufacturer's instructions from Solarbio (BC0205, BC0220, and BC0170, respectively). The samples were collected from mock- or heat-treated *R. moulmainense* leaves at 4 HAH or 7 DAH. Each biological replicate involved pooled samples from 3 individual plants, and a total of 3 biological replicates were conducted.

### RNA extraction and library preparation

TRIzol reagent (Transgene, ET121-01, Beijing, China) was used for total RNA extraction from *R. moulmainense* tissues, following the manufacturer's protocols. Library preparation and Illumina sequencing were carried out by Shanghai Majorbio Bio-pharm Biotechnology (Shanghai, China) using the Illumina Novaseq6000 platform.

For the sRNA-seq library, the Illumina TruSeq Small RNA Kit was employed for sRNA library construction. Subsequently, the Illumina HiSeq2500 platform was used to sequence the pooled sRNA libraries (Shanghai Majorbio Bio-pharm Biotechnology), generating 18–32 nt double-end reads.

### mRNA-seq data processing

Clean data (reads) were acquired by trimming all raw mRNA-seq reads that passed the FastQC quality control steps. The reads were then mapped to the *Rhododendron ovatum* genome (Wang et al. 2021a) using the Hisat2 software (http://ccb.jhu.edu/software/hisat2/index.shtml) (Kim et al. 2015). Subsequent analysis focused solely on reads that mapped to unique positions.

### sRNA-seq data processing

The raw sequencing data underwent filtration using the Fastx-Toolkit (http://hannonlab.cshl.edu/fastx_toolkit/), and only 18–32-nt reads were retained for further analysis. Adapter-free reads were mapped to small nuclear RNA, small nucleolar RNA, tRNA, and ribosomal RNA sequences using Bowtie (http://bowtie-bio.sourceforge.net/index.shtml) (Langmead and Salzberg 2012). Reads per million mapped reads (RPM) values were then assessed for normalization. To compare sRNA abundance between mock and heat treatments, DESeq2 (http://bioconductor.org/packages/stats/bioc/DESeq2/) (Love et al. 2014) was employed.

### Reverse transcription quantitative polymerase chain reaction (RT-qPCR) assay

The total RNA was extracted as above. First-strand cDNA was synthesized using 2 μg total RNA per 20 μL reaction following the manufacturer's protocols (Aidlab, PC7002, Beijing, China). RT-qPCR was performed using a FastSYBR reaction kit as instructed (CWBIO, Beijing, China). Primers for RT-qPCR were designed using NCBI Primer-BLAST software and are listed in Suppl. Table S2. The RT-qPCR data were analyzed using the 2^−ΔΔCT^ method (Livak and Schmittgen 2001). Statistical differences between treatments were determined using two-tailed Student’s *t*-test. All experiments were performed with at least three biological replicates per treatment with similar results.

### Identification of alternative splicing (AS) genes and events

For each sample, only transcripts identified in all three replicates were retained. rMATS software (v 4.0.2, http://rnaseq-mats.sourceforge.net/index.html) (Shen et al. 2014) with default parameters was used to identify AS genes, AS type, and AS number based on the assembled transcripts.

### Prediction of miRNA target genes

Prediction of the miRNA target genes was conducted using the RNAHybrid (Rehmsmeier et al. 2004) and psRobot (Wu et al. 2012).

### Population structure and diversity analyses

The principal component analysis (PCA) analysis was performed to explore the variation of transcriptomic data among individuals.

### Differential gene expression and enrichment analysis

The EdgeR software (Robinson et al. 2010) was utilized to normalize gene expression levels and identify significant DEGs (*P*_adjust_ < 0.05 and fold-change ≥ 2). For the Gene Ontology (GO) enrichment analyses, agriGO v2.0 (https://github.com/tanghaibao/GOatools) (Tian et al. 2017) was employed. Each gene set was compared against the entire set of genes in the *R. ovatum* genome (Wang et al. 2021a) as background. GO terms with a FDR of < 0.05 were considered significantly enriched.

## Results

### Transcriptome analysis of essential regulators in *Rhododendron moulmainense* thermotolerance

**Fig. 1:**
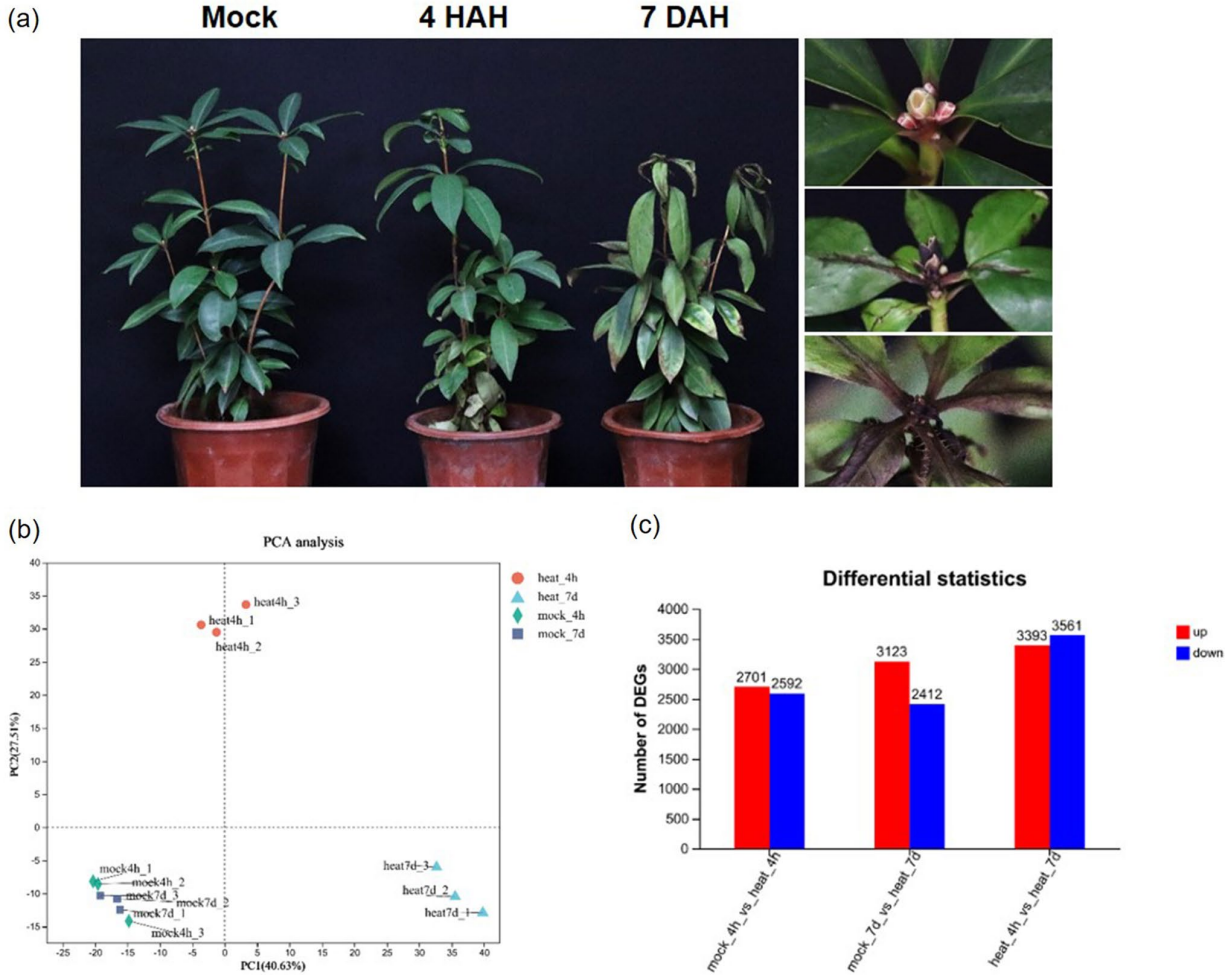
Transcriptome analysis of essential regulators in *Rhododendron moulmainense* thermotolerance. **a** The phenotype of *R. moulmainense* under heat stress at 4 h (4 HAH) and 7 days (7 DAH). **b** The two-dimensional plot of PCA representation based on RPKMs for all genes in the 12 samples. The color-coded points denote different time points and treatments: 4 h after heat treatment (4 HAH, orange), 7 days after heat treatment (7 DAH, blue), 4-h mock treated (green), and 7-day mock treated (gray). **c** The number of heat-responsive DEGs in *R. moulmainense* leaves across different treatments

Plants have developed intricate and varied strategies to withstand and cope with elevated ambient temperatures, and multiple factors contribute to their thermotolerance. Therefore, exploring the transcriptome profiles of Rhododendron plants' thermotolerance can offer valuable insights into a comprehensive molecular understanding of diverse physiological processes involved in plant introduction and transplantation. To identify crucial factors involved in the rapid response to short- and long-term heat stress in *Rhododendron moulmainense*, we subjected the plant to a 42 °C treatment. Subsequently, we collected *R. moulmainense* samples at 4 h (HAH—Hours After Heat stress) and 7 days (DAH—Days After Heat stress). As the heat stress duration extended, noticeable changes were observed: the leaves gradually drooped, the veins appeared black with necrosis, and the top leaf buds turned brown (Fig. 1a).

To investigate the alterations in the transcriptomes of *R. moulmainense* related to thermotolerance under 42 °C treatment, we conducted Illumina RNA sequencing. Leaf samples were collected from *R. moulmainense* treated at 42 °C for 4 h or 7 days, and total RNA was then extracted. *R. moulmainense* cultivated at 25 °C served as the control group. The total cDNA libraries were subjected to Illumina RNA sequencing. For mapping, we utilized a total of 76 Gb reference transcripts of *Rhododendron ovatum*, which belongs to the same subgenus as *R. moulmainense* (Wang et al. 2021a). The raw HiSeq reads were filtered and trimmed using the Illumina pipeline, resulting in approximately 40 to 63 million clean paired-end reads obtained from each of the 12 libraries (Suppl. Table S3). After eliminating adapters and low-quality reads, each sample yielded more than six billion clean reads. To visually represent the variation in transcriptomic data, we conducted PCA. As depicted in Fig. 1b, there were relatively minor differences observed in the transcriptomes among all three sample replicates, and the experimental group and control group were effectively distinguished. The first principal component accounted for 40.63% of the total variance and clearly separated the different tissues. The second principal component accounted for 27.5% of the total variance (Fig. 1b).

The distribution of reads in chromosomes revealed interesting patterns. At 4 HAH, the number of reads in chromosomes 1, 5, 6, 7, and 9 decreased; while in chromosomes 4 and 8, there was a trend of reduced reads at 7 DAH. Additionally, the number of reads in chromosome 2 significantly increased both at 4 HAH and 7 DAH (Suppl. Fig. S1), indicating that heat treatment caused changes in transcriptional activity. Transcriptome analysis of both heat-treated and mock-treated *R. moulmainense* identified a total of 10,828 DEGs across all groups. Among these, 3103 differentially regulated genes did not show similarity with other sequences in databases (Suppl. Tables S4–S6), suggesting that they might be specific to *R. moulmainense* or non-protein coding sequences. The total number of DEGs was higher in plants subjected to heat stress for 7 days compared to those exposed to heat stress for 4 h. Specifically, at 7 DAH, there were a total of 5,535 DEGs (3,123 with up-regulation and 2412 with down-regulation), while at 4 HAH, 5293 DEGs were identified, comprising 2701 with up-regulation and 2592 with down-regulation. Notably, even more DEGs (6954) were found in heat-treated plants between 4 HAH and 7 DAH, including 3393 with up-regulation and 3561 with down-regulation (Fig. 1c). These findings suggest that *R. moulmainense* employs different response pathways to adapt to short- and long-term high-temperature stresses.

### Identification and analysis of DEGs under heat treatment

The GO analysis was performed to identify functional categories of DEGs under heat stress in *R. moulmainense*. Approximately, 64 and 131 GO terms (*q*-value ≤ 0.05) were significantly enriched in heat 4 h/mock 4 h and heat 7 days/mock 7 days, respectively. In both of these comparisons, the up-regulated GO terms were primarily associated with protein folding, response to temperature stimulus, and response to heat (Suppl. Fig. S2a, b). It was also observed that certain GO terms were remarkably enriched in 4 HAH plants. The DEGs with down-regulation were primarily categorized into isoprenoid binding and abscisic acid binding, while those with up-regulation were primarily distributed into cellular response to stress and stimulus, protein phosphatase inhibitor activity, and histone acetylation (Suppl. Fig. S2a). In 7 DAH plants, the top enrichment pathways were related to photosynthesis and the auxin-activated signaling pathway. Cellular homeostasis-related genes also demonstrated significant differential expression levels (Suppl. Fig. S2b).

KEGG analysis (Kanehisa and Goto 2000; Kanehisa et al. 2023) was also performed to examine the metabolic and signaling pathways responsible for heat stress response in *R. moulmainense*. Indeed, there appears to be an issue with protein folding in the ER under 4 HAH treatment, which aligns with the findings from the GO enrichment analysis. This suggests that heat treatment disrupts the protein folding pathway in the ER, resulting in protein misfolding and loss of protein function. Subsequently, many proteins may lose their functionality, potentially influencing the observed phenotypic changes. Additionally, various metabolic pathways show abnormalities, with the synthesis of different compounds severely affected. This disruption in metabolic pathways may be linked to the impaired ER protein folding function, leading to metabolic disorders. Another possibility is that there are issues with genes associated with lignin synthesis, as well as problems in fatty acid synthesis and metabolism. Moreover, the variable splicing pathway seems to be affected by high temperature, indicating that mRNA variable splicing can be influenced (Suppl. Fig. S2c). The KEGG analysis in 7 DAH plants aligns with the GO analysis and indicates enrichment in chloroplast-mediated metabolic pathways, including phenylpropanoid biosynthesis, starch and sucrose metabolism, carbon fixation in photosynthetic organisms, carotenoid biosynthesis, and plant hormone signal transduction (Suppl. Fig. S2d). Furthermore, the transcription levels of HSPs were induced in both 4 HAH and 7 DAH plants. This observation suggests that heat stress triggers the expression of HSPs, which are known to play a role in cellular protection and stress response.

**Fig. 2:**
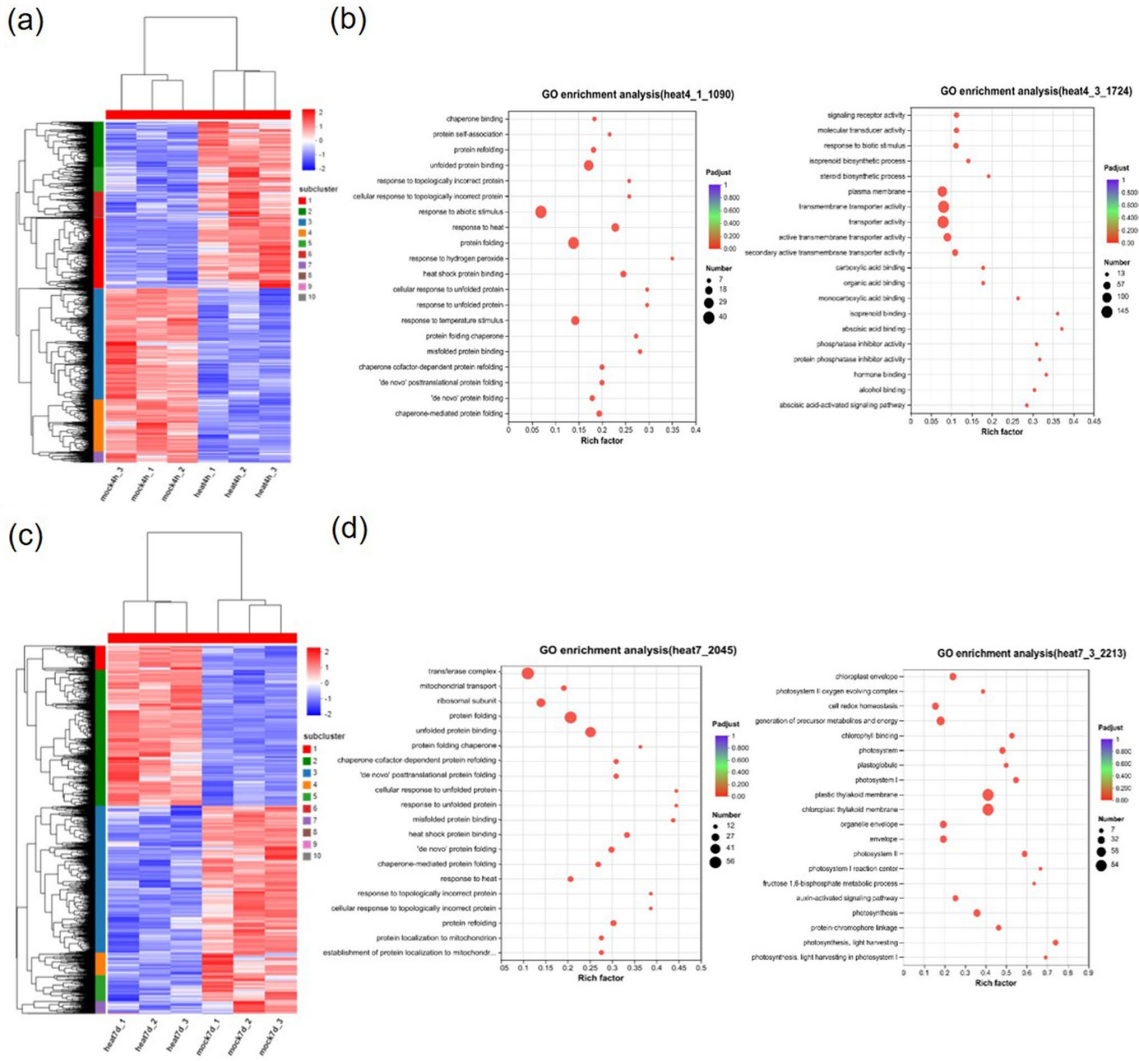
Main regulatory pathways involved in *R. moulmainense* response to heat stress. **a** Heatmap of DEGs in *R. moulmainense* leaves after heat and mock treatment for 4 h. **b** GO analysis of the 1 and 3 module genes. **c** Heatmap of DEGs in *R. moulmainense* leaves after heat and mock treatment for 7 days. **d** GO analysis of the 1 and 3 module genes. Rich Factor = the abundance or enrichment of certain transcripts in the selected transcript set compared to the entire set of transcripts in the species. Each treatment has three biological replicates

To conduct a more detailed analysis of the metabolic pathways underlying the differences in gene expression, a grouped heatmap of gene expression was generated (Fig. 2a). Subsequently, the top four subclusters in the heatmap were subjected to GO analysis to evaluate potential key regulatory genes within this module. In the “heat 4 h/mock 4 h” comparison, genes in subcluster 1 (1090) and 2 (705) exhibited significant up-regulation under short-term heat stress, while genes in subcluster 3 (1724) and 4 (398) showed significant down-regulation. Consistent with the GO analysis of the 4 h heat-treated sample, up-regulated DEGs in subcluster 1 (Fig. 2b) and 2 (Suppl. Fig. S3a) were primarily enriched in protein folding, response to heat, RNA processing, and cellular processes. On the other hand, down-regulated DEGs in subclusters 3 (Fig. 2b) and 4 (Suppl. Fig. S3a) were mainly enriched in protein transport, hormone and metabolite signaling, catalytic activity, and ATP binding.

In the "heat 7 days/mock 7 days" comparison, we also directed our attention to the top four subclusters in the heatmap (Fig. 2c). Among these subclusters, genes in subcluster 1 (360) and 2 (2045) showed significant up-regulation and were enriched in protein folding and stress response, which resembled the findings from the 4-h heat-treated plants. Meanwhile, in subclusters 3 (2213) and 4 (340), a considerable number of genes associated with photosynthesis were down-regulated. Additionally, genes involved in catalytic activity, oxidoreductase activity, chlorophyllase activity, and thylakoid membrane pathway genes were also down-regulated (Fig. 2d, Suppl. Fig. S3b). These enriched pathways may play crucial roles in responding to long-term high-temperature stress in *R. moulmainense*.

### Rapid response of protein folding to high-temperature stress

**Fig. 3:**
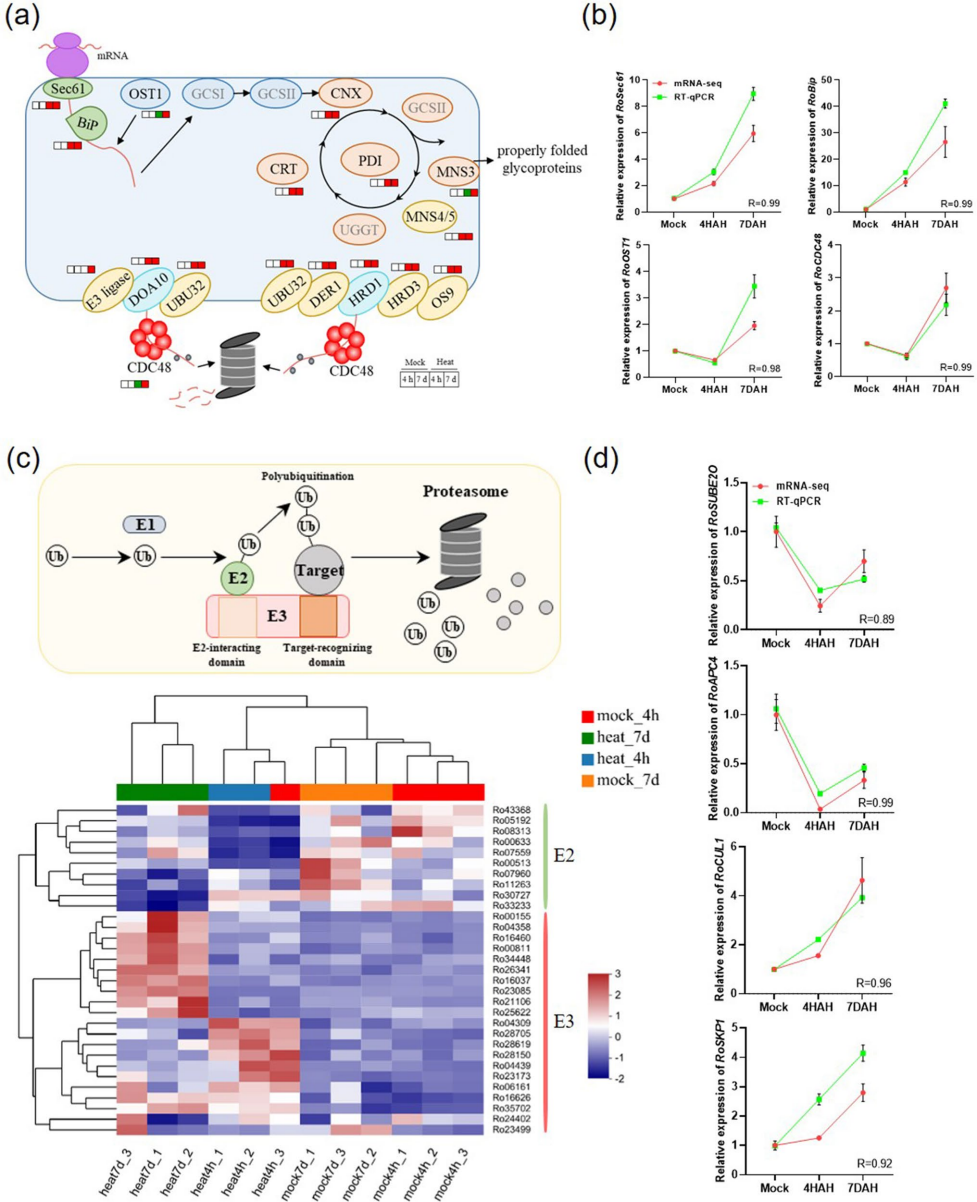
Heat stress exerts a significant impact on protein translation pathway. **a** Gene expression changes in ER-mediated protein folding during 4-h and 7-day heat treatment in *R. moulmainense*. The diagram displays relative expression changes of genes encoding different enzymes (Suppl. Table S4, S5). Red, green and white boxes represent up-regulated DEGs, down-regulated DEGs, and genes with no significant changes compared to mock-inoculated plants (adjusted *P* < 0.05), respectively. The regulation of protein homeostasis in the ER was adapted from Sun et al. (2021). **b** Verification for mRNA-seq data about ER protein folding pathway by RT-qPCR. The expression of *RoGAPDH* was used as the control. **c** The ubiquitin–proteasome system and its cellular components. The module was adapted from the KEGG website and have obtained the KEGG copyright permission (Kanehisa and Goto 2000; Kanehisa et al. 2023). The lower part shows the heatmap of DEGs related to ubiquitin–proteasome system in *R. moulmainense* leaves after heat- and mock treatment for 4 h and 7 days. **d** Verification for mRNA-seq data about ubiquitin–proteasome pathway by RT-qPCR. The expression of *RoGAPDH* was used as the control. *R* in **b** and **d** represents the correlation coefficient between the expression patterns of mRNA-seq data and RT-qPCR results, determined using the two-tailed Student’s *t*-test, and mean values ± SE, *n* = 3

One of the major adverse outcomes of heat shock is the excessive aggregation of misfolded or unfolded proteins in both the cytosol and ER. Protein degradation and folding play important roles in ER protein homeostasis (Sun et al. 2021). At 4 HAH and 7 DAH, there was a significant increase in the expression of genes associated with protein folding in the ER, indicating a need for more proteins to cope with heat stress. Moreover, the pathway responsible for degrading misfolded proteins in the ER was also activated, suggesting that attempts at protein folding were unsuccessful and led to an accumulation of misfolded proteins (Fig. 3a). At 4 HAH, *Sec61* and *Bip*, which play a role in guiding nascent proteins to translocate and fold within the ER, were remarkably up-regulated. In contrast, the expression levels of oligosaccharyltransferase (*OST1*) and *MNS3* were down-regulated. OST1 functions in transferring N-glycans (Glc3Man9GlcNAc2) to nascent proteins, and mannosidase MNS3 is responsible for modifying the mannose residues in the glycan chain. Once glycoproteins are correctly folded, they exit the ER and proceed to the Golgi apparatus (Maruyama et al. 2014). These findings suggest that the transportation of appropriately folded proteins from the ER to the Golgi was disrupted. Additionally, the transcription level of *CDC48* decreased. CDC48 acts as an AAA-ATPase, supplying the necessary energy for protein retrotranslocation to facilitate ubiquitin degradation (Begue et al. 2019). Moreover, we confirm the mRNA-seq data using the RT-qPCR method. The results showed that the expression profiles for the ER protein folding genes were highly consistent between mRNA-seq data and RT-qPCR (Fig. 3b). This indicates that a considerable number of damaged or misfolded proteins have accumulated, potentially leading to cellular toxicity.

Maintaining protein homeostasis, which involves a delicate balance between protein synthesis and degradation, is crucial for various cellular functions during plant growth, development, and stress resistance. Eukaryotic cells employ two distinct yet complementary systems—the ubiquitin–proteasome system (UPS) and autophagy—to efficiently target a wide array of proteins for degradation (Schreiber and Peter 2014). To explore whether the protein degradation pathway is involved in the heat stress response of *R. moulmainense*, heatmaps were generated to assess the expression levels of UPS- and autophagy-related genes. The UPS comprises E1 (ubiquitin-activating enzyme), E2 (ubiquitin-conjugating enzyme), and E3 (ubiquitin protein ligases) components (Zheng and Shabek 2017) (Fig. 3c). The findings demonstrated that under heat stress, most *E2s* were down-regulated, while most *E3s* were up-regulated, especially during prolonged heat stress. The RT-qPCR results also showed highly consistent expression profiles between mRNA-seq data and RT-qPCR (Fig. 3d). Additionally, SCF complex-mediated proteolysis was inhibited at 4 HAH; whereas HECT, single RING-finger type E3, and Cul-complex-mediated proteolysis were induced at 4 HAH (Fig. 3c, Suppl. Table S7).

Under heat stress, the expression level of autophagy core ATG proteins (Kim et al. 2012), such as *ATG1* (Ro37580, Ro30576), *ATG9* (Ro15215) and *ATG13* (Ro37574), were all down-regulated. The xylem's tracheary elements (TEs) function as the plant vascular system's conduits for water transport. *ATG5* can act as a regulator of TE differentiation (Kwon et al. 2010), and its expression was notably increased during heat stress (Suppl. Fig. S4). Moreover, ATG4 is a direct target for oxidation by H_2_O_2_, and in mammals, it can be activated under elevated levels of ROS (Scherz-Shouval et al. 2007). However, in *R. moulmainense*, although ROS accumulation was induced under heat stress, the expression level of *ATG4* demonstrated significant down-regulation (Suppl. Fig. S4). ATG8 typically serves as a standard marker for autophagy, and ATG4 acts as a protease responsible for eliminating additional C-terminal residues from an ATG8 precursor, thus revealing a specific glycine residue for conjugation (Mizushima and Levine 2010). Hence, the generation of autophagosomes facilitated by ATG8 may be hindered during heat stress in *R. moulmainense*. These findings suggest that the requirement for protein folding undergoes alterations under heat stress in *R. moulmainense*, and there is a pressing necessity to swiftly eliminate misfolded or impaired proteins. However, the accumulation of misfolded and damaged proteins might aggregate and negatively impact *R. moulmainense*'s adaptability.

### Spliceosome is involved in heat response

**Fig. 4:**
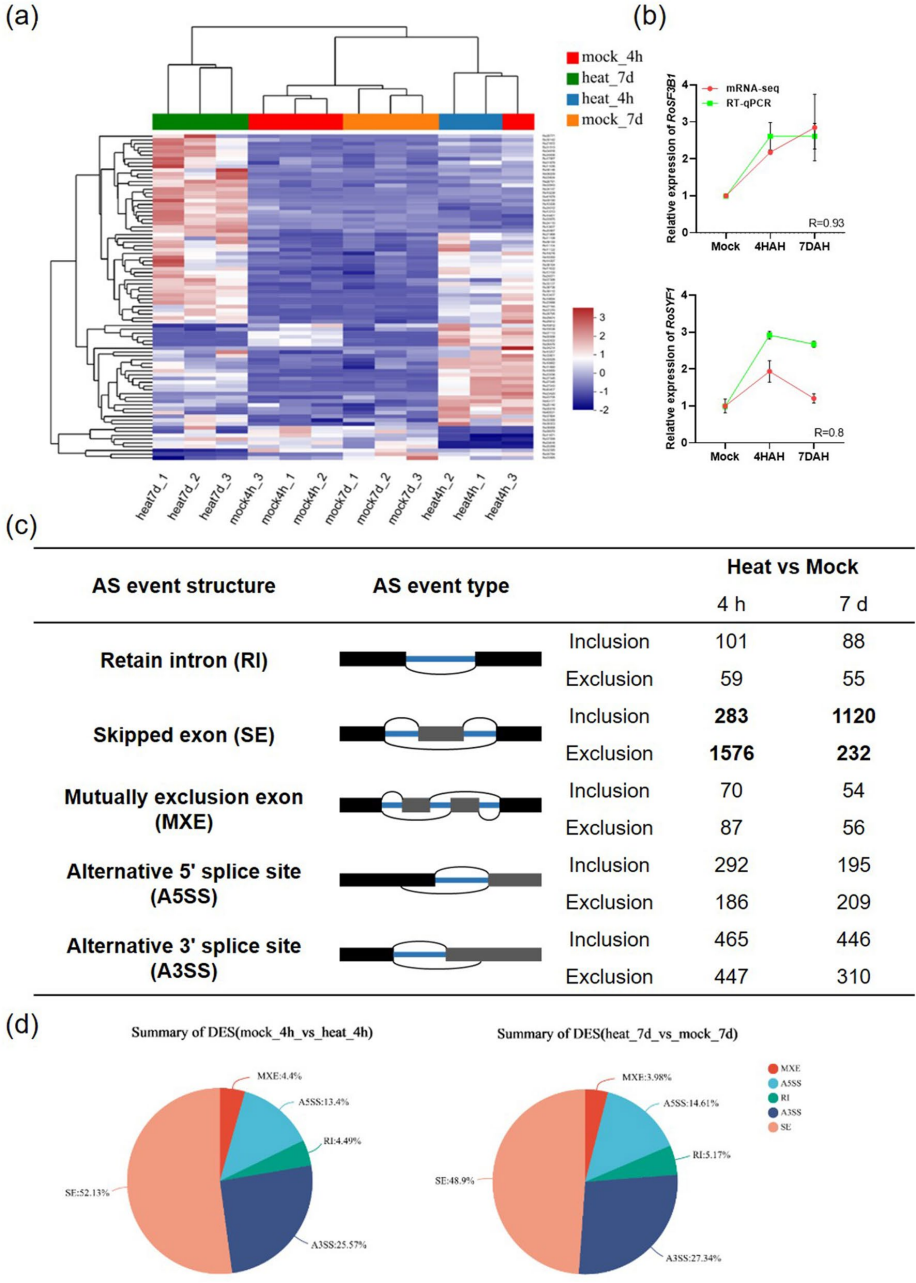
The splicing was disturbed under heat stress. **a** The heatmap of splicing pathway-related DEGs in *R. moulmainense* leaves after heat- and mock treatment for 4 h and 7 days. **b** Verification for mRNA-seq data about spliceosome pathway by RT-qPCR. The expression of *RoGAPDH* was used as the control. *R* represents the correlation coefficient between the expression patterns of mRNA-seq data and RT-qPCR results, determined using the two-tailed Student’s *t*-test, and mean values ± SE, *n* = 3. **c** Alternative splicing schematic and AS event diversity in *R. moulmainense*. The diagram illustrates AS events with black rectangles denoting flanking exons, gray rectangles indicating differential exons, thick black line representing introns, and thin black arc denotes spliced sequences. **d** The five AS event types schematic diagram and the proportion of differential AS event types under heat stress

To endure adverse conditions, plants often employ pre-mRNA splicing as a mechanism to control the expression of stress-responsive genes and reprogram intracellular regulatory networks (Dubrovina et al. 2013). The results mentioned above indicate that genes related to the spliceosome pathway were enriched during the GO analysis. The impact of heat stress on transcription leads to significant changes in cellular activities. Exposure to heat stress induces hundreds of genes (Larkindale and Vierling 2008), signifying the necessary readjustment of molecular activities required for thermotolerance. AS is a prominent mechanism governing gene expression in eukaryotes, enhancing proteome diversity while also modulating transcriptome abundance (Rosenkranz et al. 2022). AS is primarily controlled by splicing regulators, a large RNA–protein complex composed of five small nuclear RNAs (snRNAs: U1, U2, U4, U5, and U6) and hundreds of non-snRNP proteins (Reddy et al. 2013; Staiger and Brown 2013). Notably, we found remarkable changes in the expression levels of U2 small nuclear ribonucleoprotein (snRNP) components in *R. moulmainense* under heat stress (Fig. 4a, b, Suppl. Table S8). U2 snRNP belongs to the spliceosome A complex and plays a key role in recognizing the 3ʹ splice site (3ʹ SS), which is essential for pre-mRNA splicing. Furthermore, this recognition process also contributes to the regulation of variable splicing in specific gene pre-mRNA (Plaschka et al. 2018; Wan et al. 2020).

Under heat stress, the expression of *Precursor RNA processing 19* (*PRP19*) is notably up-regulated. The PRP19 complex, also referred to as the NineTeen Complex (NTC), plays a vital role in catalytically activating the spliceosome (Chan et al. 2003). As a result, spliceosome activation is induced during heat stress. Additionally, besides the core components of the spliceosome, SYF1 was identified as being involved in regulating pre-mRNA splicing efficiency (Ben-Yehuda et al. 2000). Here, we confirmed a significant increase in the expression level of *SYF1* under heat stress (Fig. 4a, b, Suppl. Table S8).

To explore whether heat stress can affect the frequency and diversity of AS events, we examined altered AS events at 4 HAH and 7 DAH (Shen et al. 2014), including alternative 5′ splice site (A5SS), alternative 3′ splice site (A3SS), retained intron (RI), mutually exclusive exons (MXE), and skipped exon (SE). The two splicing binding forms generated by these five common AS events are the inclusion isoform and exclusion isoform. We observed that the proportion of these two forms significantly changed in SE events under high-temperature conditions (Fig. 4c). Further statistical analysis revealed that SE was the most prevalent type of AS under heat stress, accounting for 52.13% and 48.9% under 4 HAH and 7 DAH, respectively. It was followed by the A3SS type (25.57%, 27.34%), A5SS type (13.40%, 14.61%), RI type (4.49%, 5.17%), and MXE type (4.4%, 3.98%) AS events (Fig. 4d).

The findings reveal that SE is the most prevalent AS event among differential splicing events. Further investigation identified specific genes with highly significant changes in SE under 4 HAH treatment: Ro05550 (*STX7*), Ro18283 (*GST*), Ro40298 (*VPS2A*), and Ro01207 (*CPRF1*). Under 7 DAH treatment, the genes with the most significant changes in SE were Ro12353 (*DGD*), Ro19919 (*AP2*), Ro12284 (*DLD*), and Ro16585 (*FBL12*). STX7 is a membrane protein that plays a crucial role in regulating cell division, hormone secretion, and plant immunity. GST and DLD are involved in regulating cellular redox homeostasis. VPS2A is a type of sugar transporter, and DGD is a sugar metabolism gene that participates in regulating plant carbon sources. CPRF1 and AP2 are both important transcription factors in plants and are involved in regulating the plant’s response to heat stress. FBL21 is an F-box protein that plays a key role in protein ubiquitination degradation. These findings reveal that the genes with significant changes in AS are actively involved in plant’s response to environmental stress and are crucial regulators of plant growth and development (Wollert et al. 2009; Mizoi et al. 2012; Sun et al. 2016; Bhatt-Wessel et al. 2018; Galle et al. 2018; Banjade et al. 2021). Therefore, alternative splicing plays a vital role in regulating the abundance of functional gene transcripts in heat adaptation of *R. moulmainense*.

### Chloroplast functions are repressed in *R. moulmainense* leaves under long-term heat stress

**Fig. 5:**
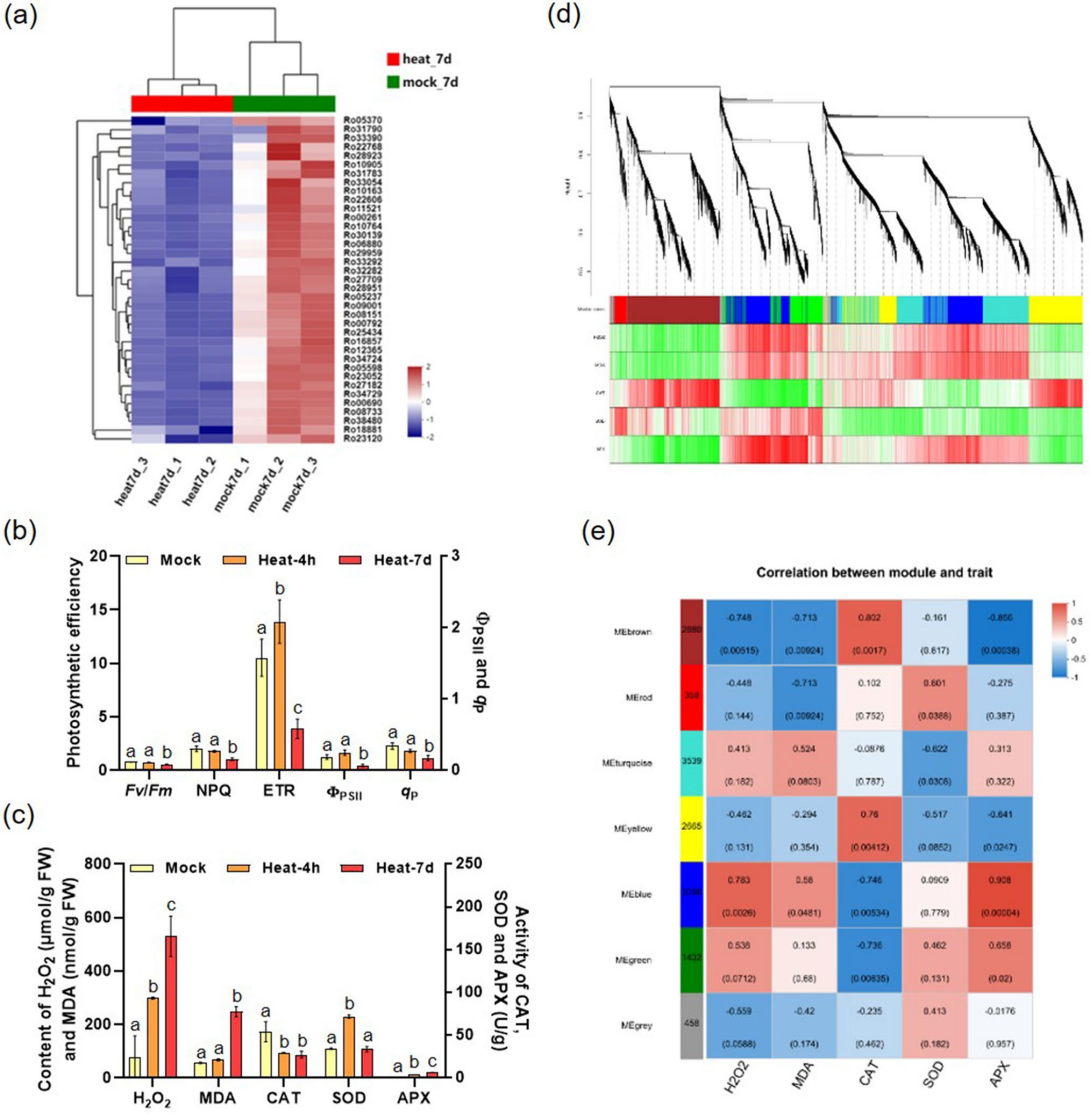
DEGs enriched in the photosynthesis pathway under 7 DAH. **a** The heatmap of photosynthesis pathway-related DEGs in *R. moulmainense* leaves after heat- and mock treatment for 4 h and 7 days. **b** Analyses of *Fv*/*Fm*, Φ_PSII_, *q*_P_, and ETR in the mock- and heat-treated *R. moulmainense* leaves at 4 HAH and 7 DAH. **c** Effects of the heat stress on reactive oxygen species (ROS) accumulation and antioxidant enzyme activity in *R. moulmainense*. Mean values ± SE, *n* = 3. Different letters above the bars in **b** and **c** indicate statistically significant differences between the treatments, determined using one-way ANOVA followed by Tukey’s multiple test (*P* < 0.05). **d, e** WGCNA analyses. In section d, a dendrogram of gene clusters based on topological overlap is shown, with modules represented by assigned colors. Each branch on the tree represents a module, and each leaf on the branch corresponds to a single gene. In section e, the associations between modules and phenotypic traits are displayed. Each row indicates a module, and the number of genes in each module is indicated on the left. Each column indicates a specific phenotypic trait, labeled below. The cells at the row–column intersection contain the correlation coefficient (|*r*|, 0.5 not shown) and the *P*-value (in brackets) for the correlation between that module and the respective trait. Dark red indicates a highly positive correlation between a specific module and a trait, while blue indicates a negative correlation. The gray module is reserved for genes that do not fall into any specific module

The chloroplast occupies a central role in oxygenic photosynthesis and primary metabolism, and beyond these functions, it has become a critical controller of plant responses to abiotic/biotic stress conditions (Zhang et al. 2020; Song et al. 2021). The photosynthetic system is composed of photosystems I and II, F-type ATPase, photosynthetic electron transport, and the cytochrome b6/f complex (Allen et al. 2011). Our results showed that most genes related to these five photosynthetic complexes were significantly down-regulated after 7 DAH treatment. Among them, the expression levels of all components of photosynthetic electron transport—*PetE*, *PetF*, *PetH*, and *PetJ*—were significantly repressed under heat stress (Fig. 5a, Suppl. Fig. S5, Suppl. Table S9). Considering that the expression levels of photosynthesis pathway-related genes were intensively interfered under 7 DAH, we determined the photosynthetic efficiency of *R. moulmainense* under 4-h and 7-day heat stress. The findings indicated that the efficiency of PSII (*Fv*/*Fm*), electron transfer rate (ETR), non-photochemical quenching (NPQ), quantum yield of PSII electron transport (Φ_PSII_), and photochemical quenching coefficient (*q*_P_) did not significantly change under 4 HAH. Furthermore, there was an upward trend in ETR and Φ_PSII_, suggesting that short-term heat stress had a minimal impact on photosynthetic efficiency. However, at 7 DAH, *Fv*/*Fm*, NPQ, ETR, Φ_PSII_, and *q*_P_ were all significantly inhibited, indicating that the functionality of chloroplast PSII was impaired under prolonged heat stress (Fig. 5b).

The ROS are predominantly generated within the chloroplast, and elevated levels of ROS can lead to damage in chloroplast function. Under heat stress, plants generate and accumulate ROS, and the content of H_2_O_2_ and MDA significantly increased (Suppl. Table S10). Conversely, plants have evolved mechanisms to counteract ROS and bolster their heat tolerance. One such mechanism involves the activation of antioxidant enzymes like CAT, APX, and SOD, which aid in enhancing thermotolerance. In this study, we found that CAT activity was inhibited, while SOD and APX activities were enhanced during heat stress (Fig. 5c, Suppl. Table S10). To delve deeper into the genes involved in redox homeostasis response, we conducted a WGCNA (Langfelder and Horvath 2008) using DEGs obtained from plants subjected to heat and mock treatments. In total, 6 distinct modules were identified, comprising of 13,970 genes. Outliers, consisting of 458 genes, were excluded from the list and represented in the gray module (Fig. 5d, Suppl. Table S1). Moreover, we discovered modules that showed substantial correlations with the measured phenotypic traits through an examination of module-trait associations. As depicted in Fig. 5e, 2 of the 6 co-expression modules consisted of genes highly associated with 1 or 2 traits ($|r|$ ≥ 0.80, *P* $<$ 0.05), which were presented in APX-related modules (blue, brown) and CAT-related module (brown). The H_2_O_2_-related module (blue), encompassing 3,096 genes, was most remarkably associated with H_2_O_2_ accumulation. The MDA-related module (brown, red), consisting of 2880 and 358 genes, respectively, was most significantly associated with MDA content. Furthermore, there was a high correlation between gene significance and module membership in the APX activity-related (cor = 0.908, *P* < 4.44E−5) and CAT activity-related (cor = 0.802, *P* < 0.0017; Suppl. Fig. S6a, b) modules.

To investigate the regulatory genes within this module, the co-expression network of the top 30 highly correlated genes was analyzed (Suppl. Fig. S6c, d). In the blue module, four genes—Ro25944, Ro05442, Ro00811, and Ro03969—exhibited the highest correlation coefficients among the 30 genes, suggesting that they might play crucial roles in H_2_O_2_ accumulation and APX activity during *R. moulmainense*’s response to heat stress. In the brown module, the top four genes were Ro10551, Ro16113, Ro33451, and Ro20356, which might play vital roles in CAT activity, APX activity, and H_2_O_2_ accumulation. Further analysis revealed that Ro25944 and Ro10551 were integral components of the membrane. Ro05442 encodes a transposase; Ro00811 encodes an E3 ligase DDB1 protein; Ro03969 encodes a Vitamin B6 protein that functions in photo-protection and homeostasis; Ro16113 encodes an early responsive to dehydration (ERD4) protein; Ro33451 encodes an RNA-recognition motif (RRM) transcription factor that plays important roles in plant immunity (Zhai et al. 2019); Ro20356 encodes a PnsB4 that functions in photosynthetic electron transport in photosystem I. Consistently, GO analysis indicated that the blue module was associated with RNA metabolism, RNA biosynthetic processes, and RNA splicing (Suppl. Fig. S6e). On the other hand, the brown module was enriched in GO categories related to the photosynthetic system, photosynthetic membrane, and cellular carbohydrate and polysaccharide metabolic processes (Suppl. Fig. S6f). These results suggest that high temperature causes significant damage to chloroplast structure, redox homeostasis, protein degradation, and plant broad-spectrum resistance.

The chloroplast serves as the primary site for synthesizing plant carbon compounds. Photosystems I and II utilize light energy to produce a proton motive force and reduce power (NADPH or NADH). ATP synthase utilizes the proton motive force to generate ATP, and both NAD(P)H and ATP are then employed for carbon dioxide fixation. According to the RNA-seq data, the expression levels of genes related to ATP synthases, such as *ATPγ, ATPδ*, and *ATPb,* were down-regulated. Consequently, we further investigated whether the impaired photosynthetic efficiency affected pathways related to photosynthetic products. As anticipated, a majority of genes responsible for starch and sucrose metabolism were down-regulated (Suppl. Fig. S7a). Products derived from photosynthetic metabolism, including glucose and ROS, are closely linked to the plant's circadian clock. Glucose has been shown to provide metabolic feedback to the circadian oscillator (Haydon et al. 2013; Roman et al. 2021). In this study, we found that circadian rhythm-related genes were also down-regulated, especially during prolonged heat treatment (Suppl. Fig. S7b).

Apart from ROS, chloroplasts also generate other stress-related signaling molecules including, reactive sulfur species, reactive nitrogen species, reactive carbonyl species, secondary metabolites, volatile compounds, and stress hormone precursors such as abscisic acid (ABA), jasmonic acid (JA), and salicylic acid (SA) (Li and Kim 2022). Our heatmap analysis indicated that genes associated with the biosynthesis and signaling of IAA, ABA, and cytokinin hormones were down-regulated (Suppl. Fig. S8). Moreover, we observed that brassinosteroid (BR) and SA-related genes were down-regulated at 4 HAH, but up-regulated at 7 DAH (Suppl. Table S4, S5). As ABA is a central regulator of plant stress defense, our results showed that genes related to the ABA binding pathway, such as *PYL* and *ABF*, were significantly down-regulated. Moreover, we found that the stomatal apertures (the ratio of width to length) decreased significantly following ABA treatment under heat stress (Suppl. Fig. S9). Additionally, MYB transcription factors are known to function in an ABA-dependent manner (Verma et al. 2016), and we observed that the majority of *MYB* family members were down-regulated after heat treatment (Suppl. Fig. S10). However, most *NAC* transcription factor family genes were up-regulated under heat stress, suggesting their potential positive roles in ABA biosynthesis (Suppl. Fig. S11).

### Small RNA regulatory pathways in *R. moulmainense* under heat stress

**Fig. 6:**
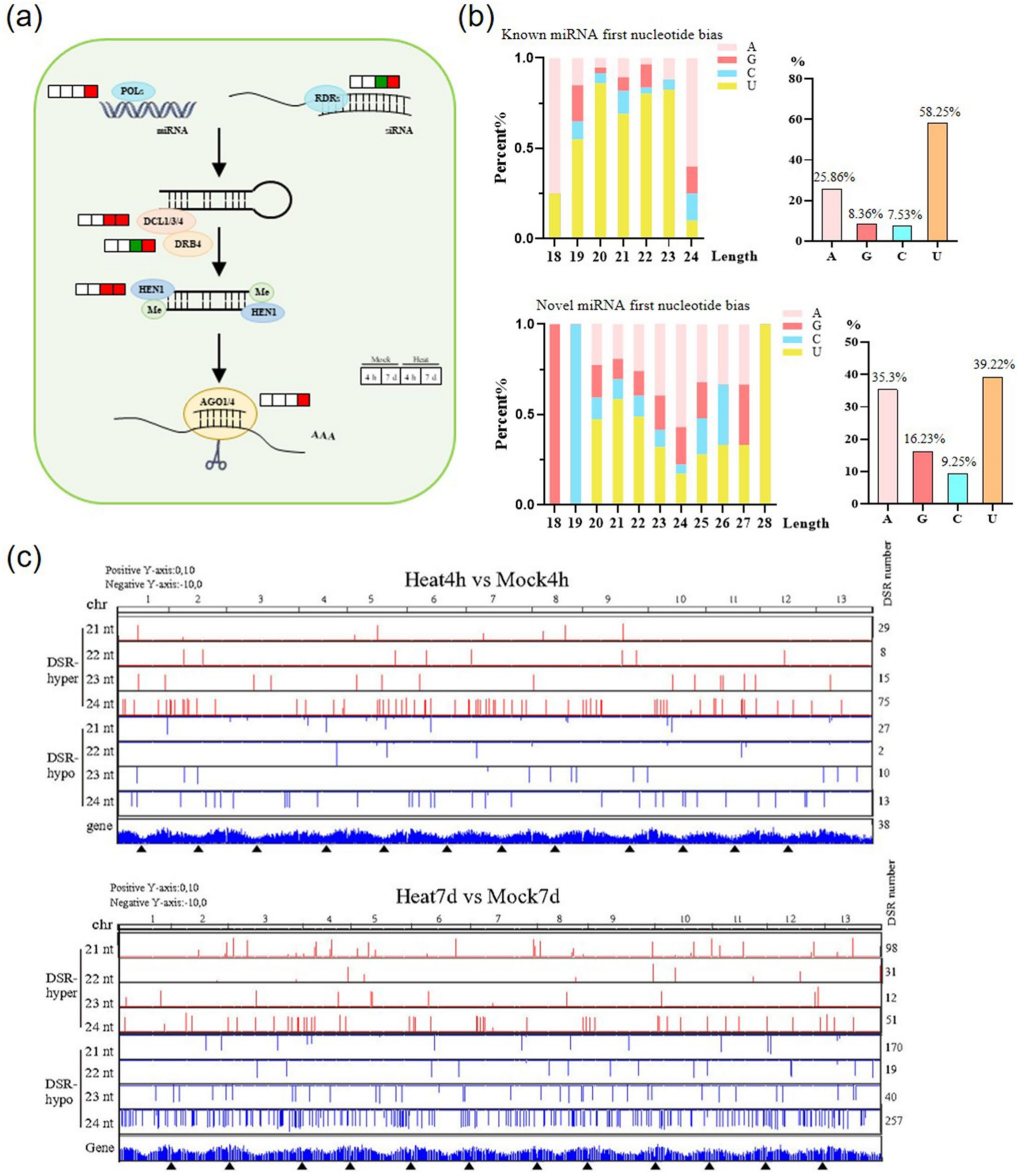
The sRNAs participate in high-temperature response in *R. moulmainense*. **a** Gene expression changes in sRNA biosynthesis during 4-h and 7-day heat treatment in *R. moulmainense*. The diagram displays relative expression changes of genes encoding various enzymes (Suppl. Table S2, S3). Red, green, and white boxes represent up-regulated DEGs, down-regulated DEGs and genes with no significant changes compared to mock-inoculated plants (adjusted *P* < 0.05), respectively. The regulation figure was adapted from Pumplin and co-workers (Pumplin and Voinnet 2013). **b** Comparison of the 5′ first nucleotide of known and novel miRNAs in *R. moulmainense*. **c** Distribution of differentially expressed miRNAs along the *R. moulmainense* genome after heat stress for 4 h and 7 days. The genome was segmented into 500-bp intervals for analysis. The arrowheads denote the position of centromeres. hypo, down-regulated; hyper, up-regulated; Chr, Chromosome

In addition to coding genes, numerous long non-coding RNAs and sRNAs, particularly miRNAs, have been identified to be crucial players in plant growth and development (Zuo et al. 2021). We conducted a study to predict candidate genes involved in sRNA biogenesis and regulatory pathways. We further examined their expression levels in RNA-seq data under both mock and heat-treated conditions. Our findings revealed that genes related to sRNA biosynthesis pathways were significantly up-regulated during heat stress (Fig. 6a, Suppl. Fig. S12) (Pumplin and Voinnet 2013), suggesting the existence of sRNA regulatory pathways in *R. moulmainense* that potentially influence its thermotolerance.

Considering the strong relationship between the length and the first nucleotide at the 5′ end of miRNAs and their interactions with associated AGO proteins, we conducted an analysis of the 5′ first nucleotide of both known and novel miRNAs. The findings indicated that 58.25% of known miRNAs began with "U" at the 5′ end, which is the preferred start for AGO1 (Fig. 6b). On the other hand, 39.22% of novel miRNAs started with “U” and 35.3% started with “A”, which is preferred by AGO2 (Fig. 6b). These data indicate that both AGO1 and AGO2 play significant roles in the miRNA-mediated processes of target transcript cleavage and translation repression in *R. moulmainense*.

To identify miRNAs that are differentially expressed in response to heat stress, we divided the *R. moulmainense* genome into consecutive, non-overlapping 500-bp windows and compared the normalized miRNA read counts in each window between the control and heat-treated samples. Notably, we focused on the abundance of miRNA size classes ranging from 18 to 24 nt at 4 HAH and 7 DAH in each 500-bp window. We observed similar numbers of hypo-differential miRNA regions (DSRs) and hyper-DSRs in the 4 HAH samples. However, in the 7 DAH tissues, there were more hypo-DSRs than hyper-DSRs (Fig. 6c). Notably, we found that the distribution of 24-nt hypo-DSRs at 7 DAH coincided with the gene distribution pattern of *R. moulmainense* chromosomes. This suggests that 24-nt miRNAs may play pivotal roles in modulating stable gene expression during prolonged heat stress in *R. moulmainense*.

### MiRNAs are involved in *R. moulmainense* thermotolerance

**Fig. 7:**
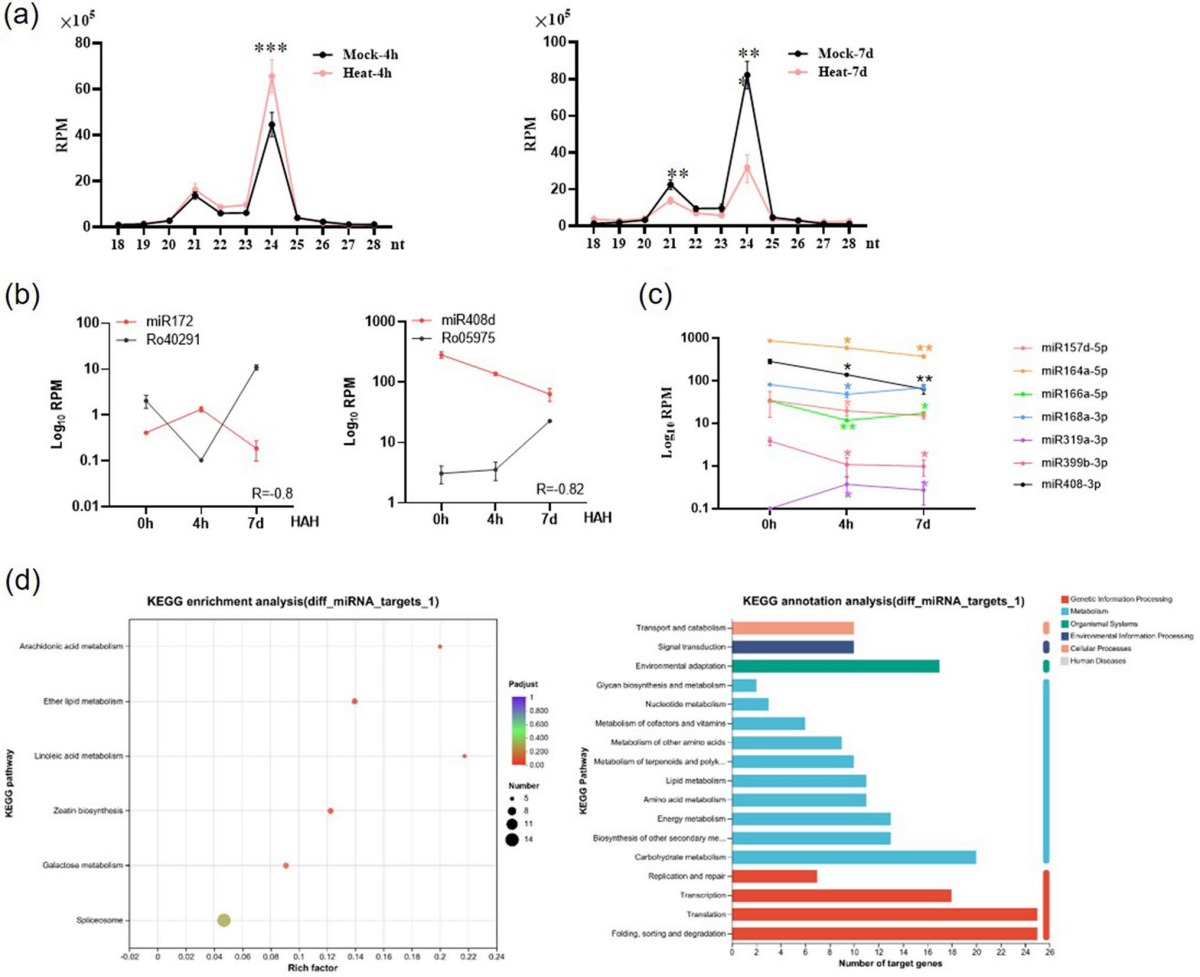
MiRNAs play a crucial role in *R. moulmainense* thermotolerance. **a** Nucleotide size distribution of miRNAs from *R. moulmainense* leaves at 4 HAH and 7 DAH. **b** Expression patterns of miR172 and miR408d, along with their target genes. The y-axis represents the log_10_ transformation of RPM and RPKM for miRNAs and genes, respectively. *R* represents the negative correlation coefficient between the expression patterns of miRNAs and their predicted target genes, determined using the two-tailed Student’s *t*-test, and mean values ± SE, *n* = 3. **c** Identification of differentially expressed miRNAs in *R. moulmainense* at both 4 HAH and 7 DAH. **d** KEGG enrichment and annotation analysis of target genes regulated by differentially expressed miRNAs in *R. moulmainense*. Mean values ± SE, *n* = 3. Asterisks in **a** and **c** indicate significant differences between treatments (*P < 0.05, **P < 0.01, ***P < 0.001), determined using the two-tailed Student’s *t*-test

To gain a deeper understanding of the role of miRNAs in *R. moulmainense* transcriptomic responses to heat stress, we constructed and sequenced miRNA libraries, resulting in 4–8 million mapped reads for each sample (Suppl. Table S11). MiRNAs are one of the major classes of sRNAs, and play vital roles in regulating plant thermotolerance and high-temperature acclimation (Zuo et al. 2021). In the 4-h mock and heat-treated samples, the miRNA populations were characterized by a prominent size of 24 nt. However, under 7 days of treatment, the 21-nt and 24-nt classes of miRNAs were significantly reduced (Fig. 7a). Further analysis revealed that miR172 (sequence: GGAAUCUUGAUGAUGCUGCAGCAG) was induced under 4-h heat treatment but reduced under 7-day heat treatment. The target gene of miR172 was *AP2*, and interestingly, we observed that the expression level of *AP2* (Ro40291) was down-regulated under 4-h heat treatment and up-regulated under 7-day heat treatment (Fig. 7b).

Moreover, we conducted a more detailed analysis of differentially expressed miRNAs. Specifically, miR157, miR164, miR166, miR168, miR399, and miR408 exhibited significant changes at both 4 HAH and 7 DAH (Fig. 7c, Suppl. Table S12, S13). Their predicted conserved targets were found to be involved in crucial processes related to metabolism and environmental adaptability (Suppl. Fig. S13). To gain a deeper understanding of the regulatory roles of these miRNAs in *R. moulmainense*, we conducted a KEGG analysis for all the predicted target genes. Most of these genes were associated with important functions such as plant signal transduction, environmental adaptation, carbohydrate metabolism, and protein folding, sorting, and degradation (Fig. 7d). These findings strongly suggest that the miRNA targets in *R. moulmainense* play essential roles in modulating proper development and enabling effective adaptation to the environment in this species.

Given the higher number of differentially expressed miRNAs observed at 7 DAH, we delved deeper into analyzing the potential targets of these miRNAs. The protein interaction network of the target genes revealed several core factors with higher degrees, including Ro38362 (MYB), Ro22879 (SPL), Ro25312 (CYCA), Ro19745 (HSP), and Ro43413 (ARF). It is worth noting that MYB, SPL, and ARF are all regulated by known miRNAs (Suppl. Table S1). Of particular interest, we found that *MYB* is one of the transcription factors that undergoes the most significant changes in response to heat stress in *R. moulmainense* (Suppl. Fig. S14). Additionally, MYB appears to have an interaction with the SPL9 transcription factor in the network. SPL9 is known to regulate plant responses to stress by regulating ROS accumulation and activating the SA signaling pathway (Stief et al. 2014). Furthermore, ARF is predicted to have a potential association with MYB and is associated with the membrane transport protein APPN, suggesting its crucial role in the heat stress response. Cell cycle protein A (CYCA) and HSP were also predicted to be targeted by novel miRNAs. These results collectively suggest that numerous miRNAs co-regulate the biological processes of *R. moulmainense* in response to heat stress, indicating a complex and interconnected regulatory network at play.

## Discussion

As an alpine evergreen azalea, *R. moulmainense* has specific requirements for its growth environment. In low-altitude environments, temperature plays a crucial role, and the potential mechanism by which *R. moulmainense* responds to high-temperature stress is still elusive. This species holds significant observational, food and medicine value and occupies an important evolutionary status within the genus *Rhododendron*. Herein, we provided a high-quality reference genome assembly for *R. moulmainense*, which serves as a crucial reference point for further research on this species. Moreover, we conducted an in-depth analysis of *R. moulmainense*'s transcriptome and sRNAs at different time points under high-temperature stress. This allowed us to unravel the intricate miRNA–mRNA regulatory network that *R. moulmainense* employs in response to high-temperature stress. Our findings lay a solid foundation for the potential introduction of high-altitude rhododendrons into low-altitude regions, facilitating their successful cultivation and acclimation.

Numerous studies have focused on the cold resistance and ultraviolet tolerance of alpine plants (Klatt et al. 2018; Zhang et al. 2019). However, there is limited research on the stress adaptability of alpine plants when introduced to low-altitude environments. The main environmental difference between high-altitude and low-altitude regions is temperature. Currently, the regulatory mechanism of high-temperature stress adaptability in alpine azaleas remains poorly understood. Some studies on *Rhododendron hainanense* have shown that pre-heat acclimation treatment can enhance their heat resistance, with heat-induced Rubisco activation protein 1 (RCA1) playing a vital role in heat adaptation genes (Wang et al. 2020). In the case of *R. ovatum*, research has demonstrated that the transcription levels of *HSP* and *TPS* significantly increased under heat stress (Wang et al. 2021a). Our study, however, found that the ER-mediated protein folding pathway plays a key role in regulating the rapid response to high-temperature stress in *R. moulmainense*. During the 4-h heat treatment, key genes involved in protein folding were significantly up-regulated. This up-regulation may lead to the accumulation of misfolded proteins when protein folding demand surpasses the folding capacity, potentially causing aggregation and toxicity in cells (Liu and Howell 2016). Additionally, we observed a significant reduction in E2, a component of the ubiquitin degradation pathway, under heat stress. This reduction could impede the timely degradation of misfolded proteins by the 26S proteasome, possibly leading to cellular damage. Furthermore, the protein transport process was inhibited, which is critical for maintaining dynamic protein equilibrium in plant cells during development and environmental responses. Therefore, inhibiting protein transport could disrupt various cellular processes due to the coordination between different organelles and the nucleus being compromised (Sun et al. 2021), especially for organellar proteins that are encoded in the nucleus and then transported to functional organs for modification and maturation within their respective organelles.

AS is a critical process in regulating gene expression, as it enhances proteome diversity and increases transcriptome abundance AS has been shown to be a significant component of plant responses to environmental stress, leading to substantial changes in the splicing profile of numerous genes (Lin and Zhu 2021; Rosenkranz et al. 2022). For instance, in Arabidopsis, glycerol kinase undergoes AS in response to shading conditions induced by photoreceptors. This AS event results in the relocation of the protein from the chloroplast to the cytoplasm, enhancing the photorespiratory bypass and reducing light inhibition caused by fluctuations in light intensity. These proteins also exhibit light-dependent localization (Ushijima et al. 2017). AS can be considered a "molecular thermometer" in plants, enabling them to rapidly adjust the abundance of functional transcripts to adapt to environmental disturbances (Capovilla et al. 2018). In rice, the heat shock transcription factor OsHSFA2d has been identified to have two splicing variants, OsHSFA2dI and OsHSFA2dII. When plants experience heat stress, OsHSFA2d generates the transcriptionally active OsHSFA2dI through AS events, which then participates in the heat stress response (Cheng et al. 2015). However, it remains unknown whether the AS pathway is involved responsible for the heat response of high-altitude rhododendrons. In our study, we observed significant changes in the expression levels of splicing regulators, such as the *U2 complex* and *PRP19*. Additionally, we found that AS events in *R. moulmainense* were substantially altered under long-term heat stress conditions, with SE being the most prevalent type of AS event. The genes showing the most significant changes in AS primarily function as transcription factors (*CPRF1*, *AP2*) involved in heat stress response, genes related to sugar metabolism and transport (*VPS2A*, *DGD*), genes in the ubiquitin–proteasome pathway (*FBL21*), and genes related to regulating cellular redox homeostasis pathways (*GST*, *DLD*) (Wollert et al. 2009; Mizoi et al. 2012; Sun et al. 2016; Bhatt-Wessel et al. 2018; Galle et al. 2018; Banjade et al. 2021). These findings imply that AS plays a crucial role in *R. moulmainense*'s response to high-temperature stress. Therefore, further investigation is necessary to assess the expression spectrum, subcellular localization, and functions of the encoded proteins after undergoing AS. This exploration will help unravel the signal pathway mechanism of *R. moulmainense* in response to high-temperature stress.

GO analysis was conducted to determine the functional categories of DEGs in plants after 7 days of heat treatment. The results indicated that the top enriched pathways were all related to photosynthesis, suggesting that the photosynthetic organs were severely damaged and chloroplasts may have been nearly destroyed. Consistent with the GO analysis, KEGG enrichment also revealed significant disturbances in chloroplast metabolism, including carotenoid biosynthesis, starch and sucrose metabolism, and plant hormone signal transduction. As the heat treatment time increased, the efficiency of photosynthesis significantly decreased, and the expression of genes related to carbohydrate synthesis decreased as well. Furthermore, the expression of circadian rhythm regulatory factors was significantly inhibited. This may indicate that limited carbon sources could affect plant growth and development, and changes in the circadian rhythm might alter the flowering period (Haydon et al. 2013; Roman et al. 2021). In heat-acclimated *R. hainanense*, heat stress induced the expression of PET and ATP synthase-related genes, potentially leading to excessive energy consumption (Wang et al. 2020). However, in *R. moulmainense*, these genes were significantly down-regulated, suggesting that an excess accumulation of energy could also damage cells. Moreover, through WGCNA correlation analysis, six different co-expression modules were identified, two of which were highly correlated with cellular ROS homeostasis. In the selected region, DEGs related to membrane homeostasis, the ubiquitin–proteasome pathway, and photosynthetic electron transport were found. Key node genes analyzed by WGCNA were associated with plant immunity. Plant immunity factors (RRM) play an important role in regulating homeostasis. These results indicate that photosynthesis-related proteins may be potential core genes for improving heat resistance in *R. moulmainense*. Therefore, our study revealed the specific pattern of cell homeostasis regulation at the transcriptional level in high-altitude rhododendron under heat stress and provided valuable information about key genes involved in cell oxidative homeostasis and immune regulation.

In addition to functional genes, miRNA is also a key epigenetic regulatory factor for gene expression, primarily playing a crucial role at the post-transcriptional level (Zuo et al. 2021). Currently, numerous studies have unveiled the important functions of miRNA in plant growth and development. Our transcriptome analysis reveals that at 7 DAH, genes related to sRNA biogenesis pathways were significantly induced. Through sRNA sequencing technology, we discovered both known and new miRNAs that respond to high-temperature stress in high-altitude rhododendron. Among them, we identified 10 differentially expressed known miRNAs and 564 differentially expressed novel miRNAs, indicating the existence of numerous species-specific miRNAs in high-altitude rhododendron that play a crucial role in regulating high-temperature stress in *R. moulmainense*. However, the specific functions of these newly identified miRNAs and their targets require further investigation.

Transposable elements (TEs) play important roles in regulating genome stability. Reports have demonstrated that plant 24-nt sRNAs play important roles in regulating the expression of TEs through RNA direct DNA methylation (Lewsey et al. 2016; He et al. 2019). Moreover, TEs can produce miRNAs, which could have significant effects on host gene transcripts that share sequences with TEs (Lisch 2009) Here, we found that the distribution of 24-nt miRNA at 7 DAH coincided with the gene distribution pattern of *R. moulmainense* chromosomes. Therefore, it is necessary to investigate the specific loci on the chromosome where the reduced 24-nt miRNAs occur and to identify the ability and mechanism of action of 24-nt novel miRNA in regulating chromosome stability and gene expression under high-temperature stress in *R. moulmainense*.

MiR172 has been documented to play a role in the thermosensory pathway, regulating ambient temperature-responsive flowering even under non-stressful conditions. Transgenic plants overexpressing miR172 displayed early flowering that was insensitive to temperature variations (Lee et al. 2010). In both safflower leaf and rice post-meiosis panicle tissues, miR172 exhibited significant down-regulation under heat stress, while its target *AP2* genes were up-regulated, suggesting the crucial involvement of the miR172-*AP2* module in plant high-temperature response (Kouhi et al. 2020; Peng et al. 2020) In our study, we observed a significant up-regulation of miR172 during the early stage of heat stress (4 HAH), followed by a significant down-regulation at 7 DAH. Concomitantly, its predicted target gene Ro70291 (*AP2*) also displayed corresponding changes. These results suggest that the miR172-*AP2* module likely plays a conservative role in regulating changes in the flowering period under heat stress in high-altitude rhododendron. Consequently, heat stress-responsive miRNAs may result in morphological changes and physiological adaptations in *R. moulmainense* by modulating their target genes at the post-transcriptional level during heat stress.

Transcription factors have been identified as a crucial component of plant stress adaptation, playing a role in regulating the expression of stress-responsive genes through different signal activations (Ohama et al. 2017; Wang et al. 2021b). In an evolutionary study of *R. ovatum* (Wang et al. 2021a), it was found that NAC transcription factors may be key factors for regulating a wide range of stress responses, contributing to *R. ovatum*'s adaptability to diverse environments at low altitudes during evolution. In our study, we found that *NAC* is among the main transcription factors involved in differential regulation under high-temperature stress (Suppl. Fig. S15). Hence, the NAC factor may serve as a potential conservative regulator of environmental adaptation in high-altitude rhododendron, warranting further exploration of its potential downstream target genes and regulatory pathways. Additionally, in our analysis of the differential miRNA target gene interaction network, we observed that MYB, SPL, and ARF transcription factors occupy crucial core positions and display potential regulatory relationships. MYBs are closely related to ABA, our results showed that ABA synthesis and signaling pathways are significantly inhibited, and consistently, the change in stomatal aperture is not obvious under heat stress. These results underscore the essential roles of MYBs in Rhododendron’s response to heat stress. Considering that current transgenic and genome editing technologies for high-altitude rhododendron are not yet fully developed, targeted modification of functional genes presents technical challenges. Therefore, we further identified potential miRNAs (miR159, miR319-*MYB*; miR156-*SPL*; miR160-*ARF*) targeting these three types of TF families in *R. moulmainense*. These miRNAs could serve as potential factors for enhancement and domestication and may be employed in the future to improve the characteristics of *R. moulmainense* using exogenous miRNA spraying and nanomaterial delivery techniques (Liu et al. 2023).

## Conclusion

In summary, we identified the altered genes in the high-altitude evergreen rhododendron *R. moulmainense* under high-temperature stress, which could provide valuable resources for investigating the conservation and species-specific aspects of the high-altitude rhododendron genome. Additionally, we unraveled the biological processes and potential key candidate genes, along with the related regulatory miRNA–mRNA networks, involved in *R. moulmainense*'s response to high-temperature stress. These genes and miRNAs can serve as target genes for enhancing desired traits in the domestication and breeding of high-altitude rhododendrons.

### Supplementary Information

Below is the link to the electronic supplementary material.

Supplementary file1 (DOCX 3300 KB)

Supplementary file2 (XLSX 2437 KB)

## Data Availability

The *Rhododendron moulmainense* plants used in this study are reserved in the Wutong Mountain National Park, and are available on request. All the data are shown in the main manuscript and in the Supplementary Materials. The sequencing data described in this manuscript were submitted to the National Center for Biotechnology Information (NCBI) under accession codes PRJNA985096.
