# Supplementary material for: Integrated analysis of transcriptome and small RNAome reveals regulatory network of rapid and long-term response to heat stress in Rhododendron moulmainense
Source: Planta. 2024 Mar 29;259(5):104. doi: 10.1007/s00425-024-04375-5 (PMC10980653; doi:10.1007/s00425-024-04375-5)
Supplement: Supplementary file 1 — Supplementary file1 (DOCX 3300 KB) [file 425_2024_4375_MOESM1_ESM.docx]

**Supplementary Materials**

**Integrated analysis of transcriptome and small RNAome reveals regulatory network of rapid and long-term response to heat stress in** ***Rhododendron moulmainense***

Si-Jia Liu ^1^, Chang Cai ^2^, Hong-Yue Cai ^1^, Yu-Qing Bai ^3^, Ding-Yue Wang ^3^, Hua Zhang ^1^, Jin-Gen Peng ^1, *^ and Li-Juan Xie ^1, *^

^1^ College of Architectural Engineering, Shenzhen Polytechnic University, Shenzhen 518055, China

^2^ Guizhou Provincial Key Laboratory for Information Systems of Mountainous Areas and Protection of Ecological Environment, Guizhou Normal University, Guiyang 550001, China

^3^ Administrative Office of Wutong Mountain National Park, Shenzhen 518004, China

*** Corresponding author:**

Li-Juan Xie

College of Architectural Engineering, Shenzhen Polytechnic University,

Guangdong, Shenzhen 518055, P.R. China

Tel: +86-0755-26018758

Fax: +86-0755-26018406

E-mail: [xlj@szpt.edu.cn](mailto:xlj@szpt.edu.cn)

Jin-Gen Peng

College of Architectural Engineering, Shenzhen Polytechnic University,

Guangdong, Shenzhen 518055, P.R. China

Tel: +86-0755-26019267

Fax: +86-0755-26018406

E-mail: [pengjingen@szpt.edu.cn](mailto:pengjingen@szpt.edu.cn)


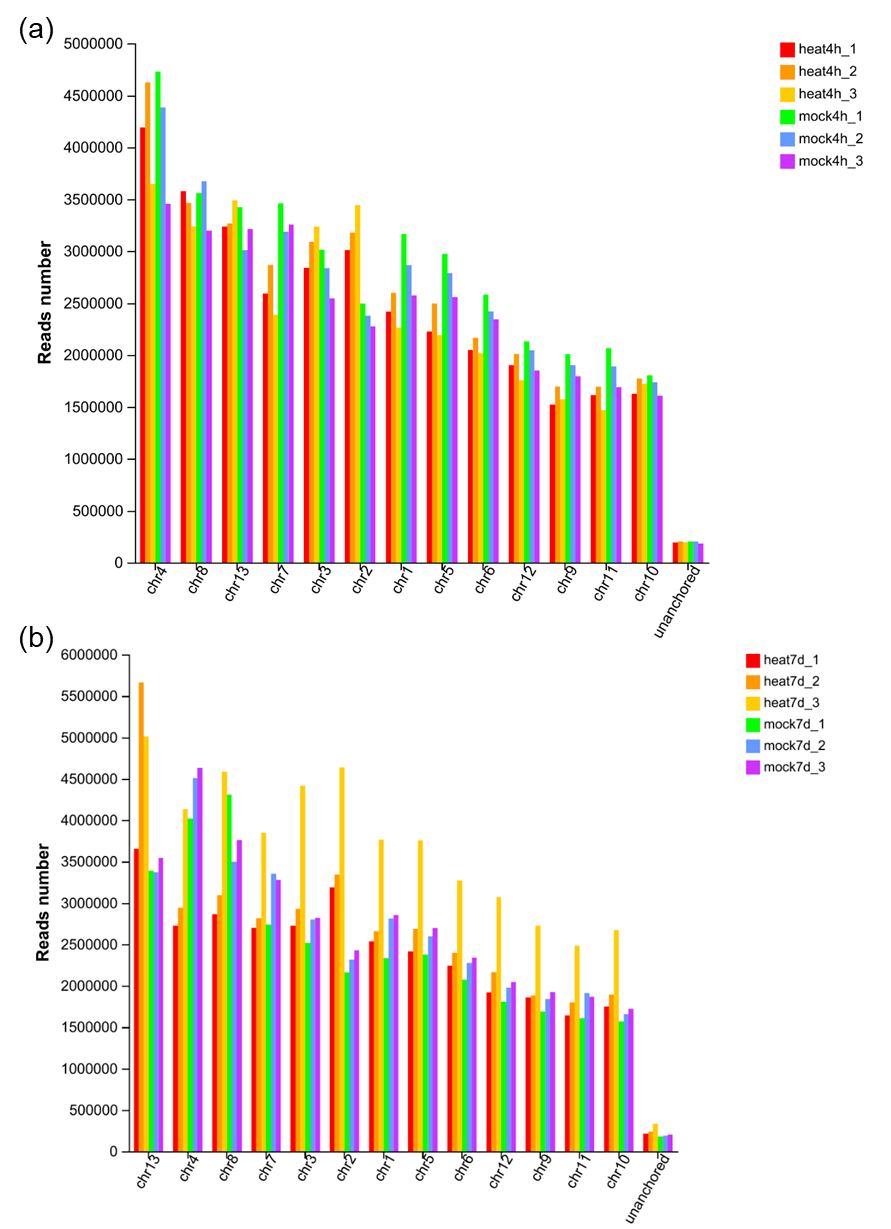


**Suppl. Fig. S1** Reads distribution in chromosomes under heat stress. **a** Reads distribution in chromosomes under 4 HAH. **b** Reads distribution in chromosomes under 7 DAH.


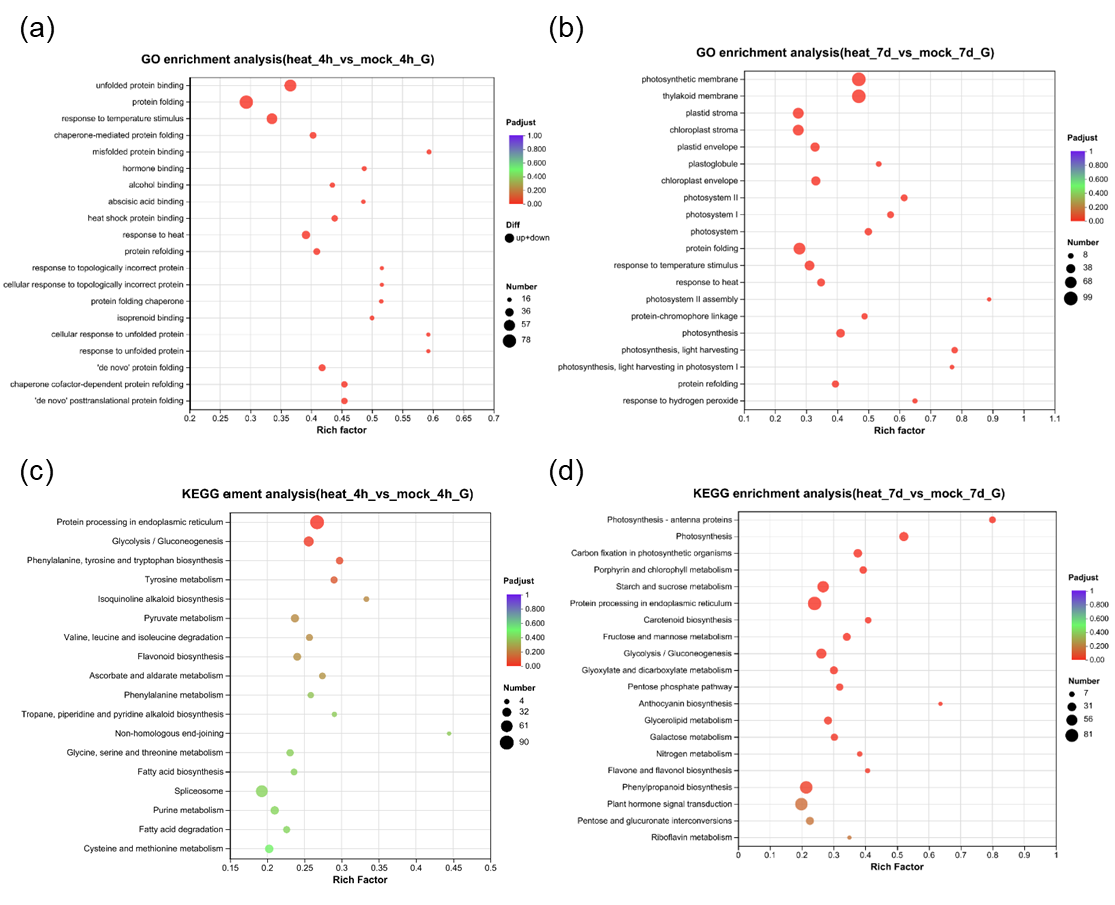


**Suppl. Fig. S2** GO and KEGG enrichment analyses of gene modules under 4 HAH and 7 HAH. **a** GO analysis of the DEGs in *R. moulmainense* leaves after heat and mock treatment for 4 hours. **b** KEGG analysis of the DEGs in *R. moulmainense* leaves after heat and mock treatment for 4 hours. **c** GO analysis of the DEGs in *R. moulmainense* leaves after heat and mock treatment for 7 days. **d** KEGG analysis of the DEGs in *R. moulmainense* leaves after heat and mock treatment for 7 days. Rich Factor = the abundance or enrichment of certain transcripts in the selected transcript set compared to the entire set of transcripts in the species.


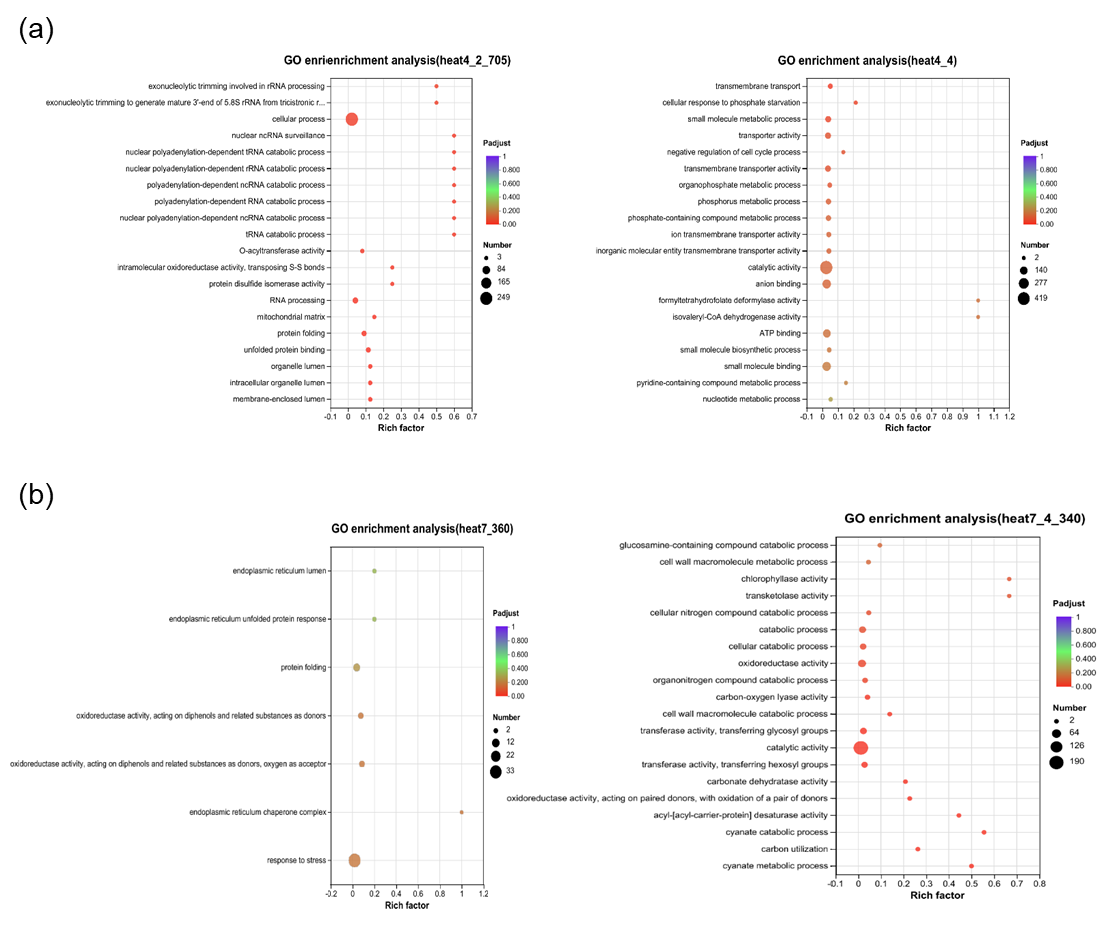


**Suppl. Fig. S3** GO and KEGG enrichment analyses of gene modules at 4 HAH and 7 DAH. **a** GO analysis of the 2 and 4 module genes from 4 HAH DEGs. **b** GO analysis of the 2 and 4 module genes from 7 DAH DEGs. Rich Factor = the abundance or enrichment of certain transcripts in the selected transcript set compared to the entire set of transcripts in the species. Each treatment has three biological replicates.


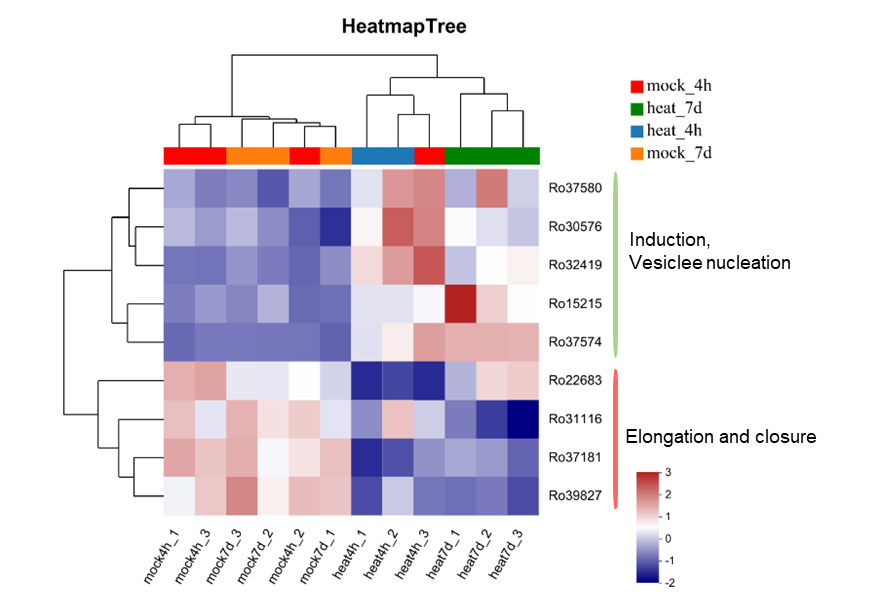


**Suppl. Fig. S4** Heatmap of autophagy pathway-related DEGs under heat stress. The heatmap of autophagy pathway-related DEGs in *R. moulmainense* leaves after heat and mock treatment for 4 hours and 7 days.


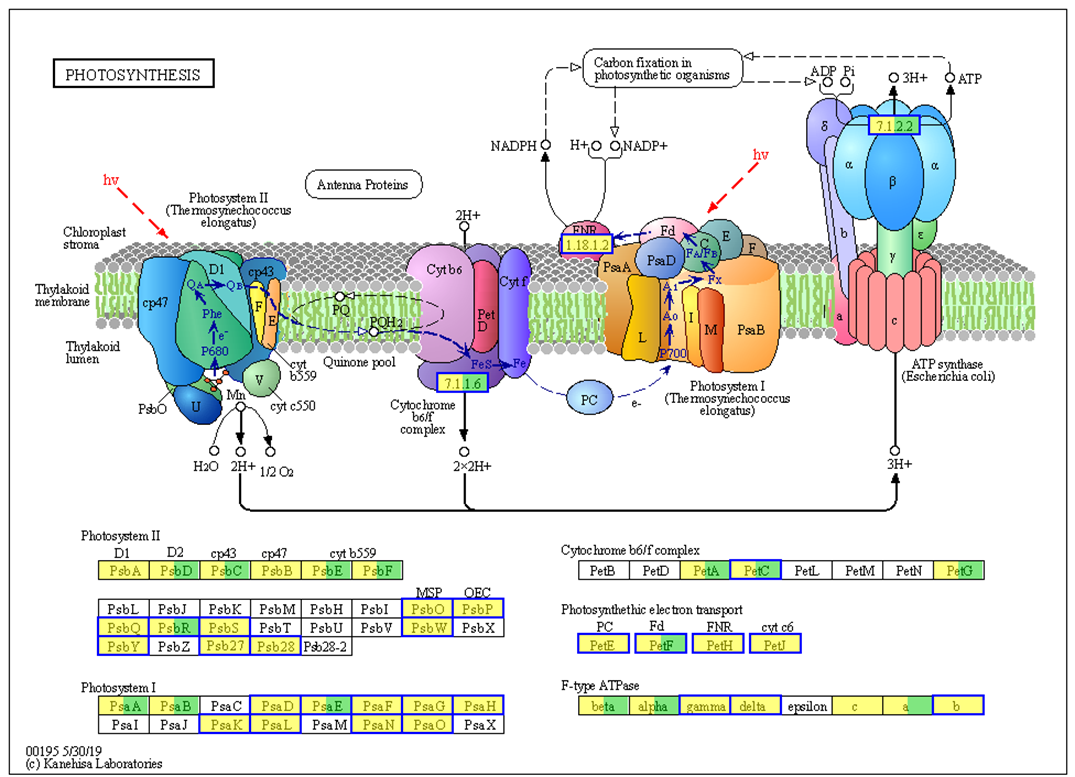


**Suppl. Fig. S5** DEGs enriched in the photosynthesis pathway at 7 DAH. The map of the photosynthesis pathway was obtained from the KEGG database (map00195) and have obtained the KEGG copyright permission (Kanehisa and Goto 2000; Kanehisa et al. 2023). DEGs enriched in the map were divided into two parts (left and right) with colors representing known and new gene families, respectively. The blue border represents a significant downregulation of gene expression levels.


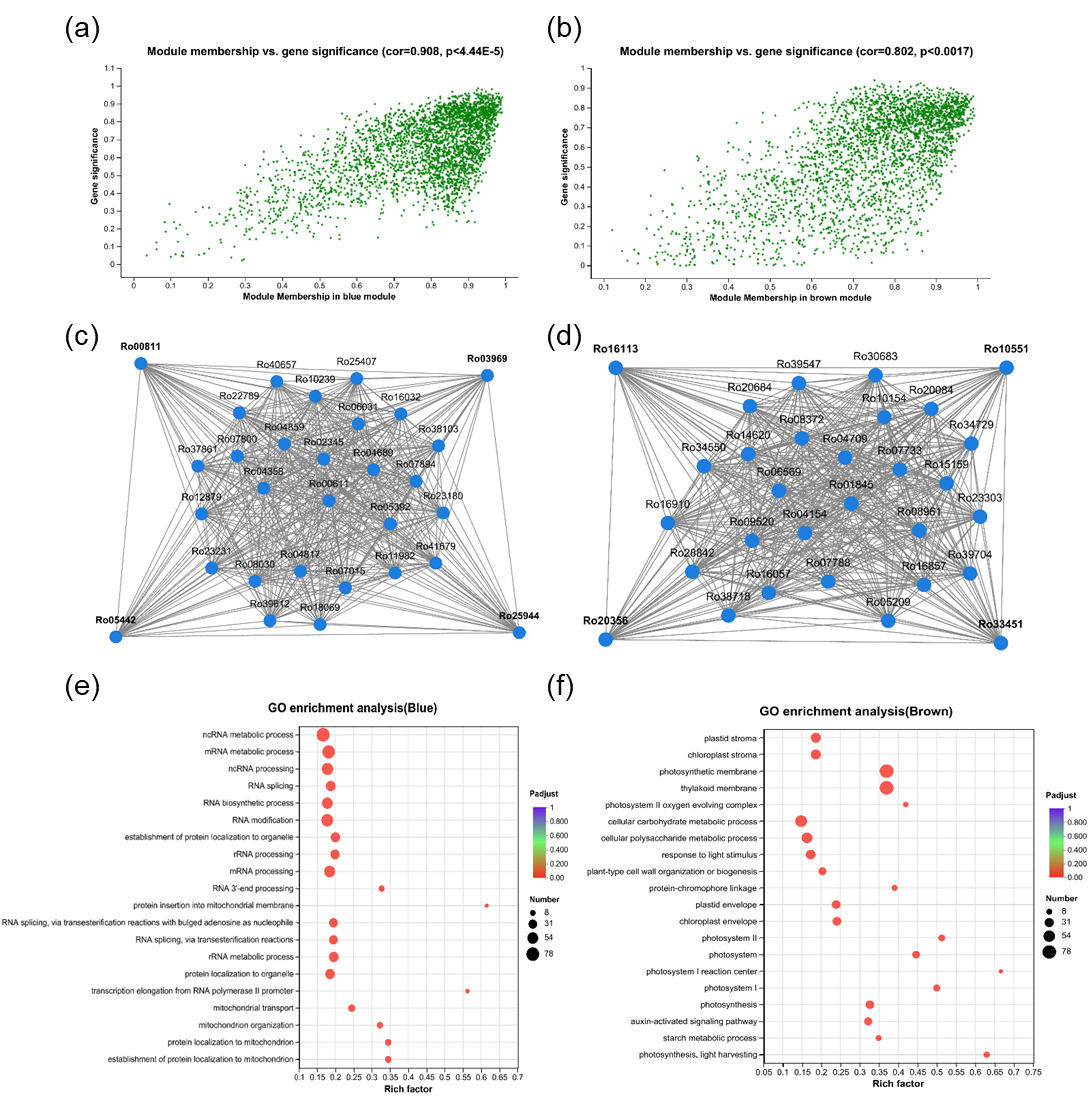


**Suppl. Fig. S6** The scatterplot and gene co-expression network analysis of heat vs. mock treatment. **a** Heat vs. mock in the H_2_O_2_ accumulation and APX enzyme activity related module (blue). **b** Heat vs. mock in the CAT enzyme activity related module (brown). **c** The H_2_O_2_ accumulation and APX enzyme activity-related genes co-expression network. **d** The CAT enzyme activity-related genes co-expression network. Highly significant correlations were found between heat and mock treatments in these two modules. **e** The heatmap of expression in APX activity related module (blue). **f** The heatmap of expression in CAT activity related module (brown).


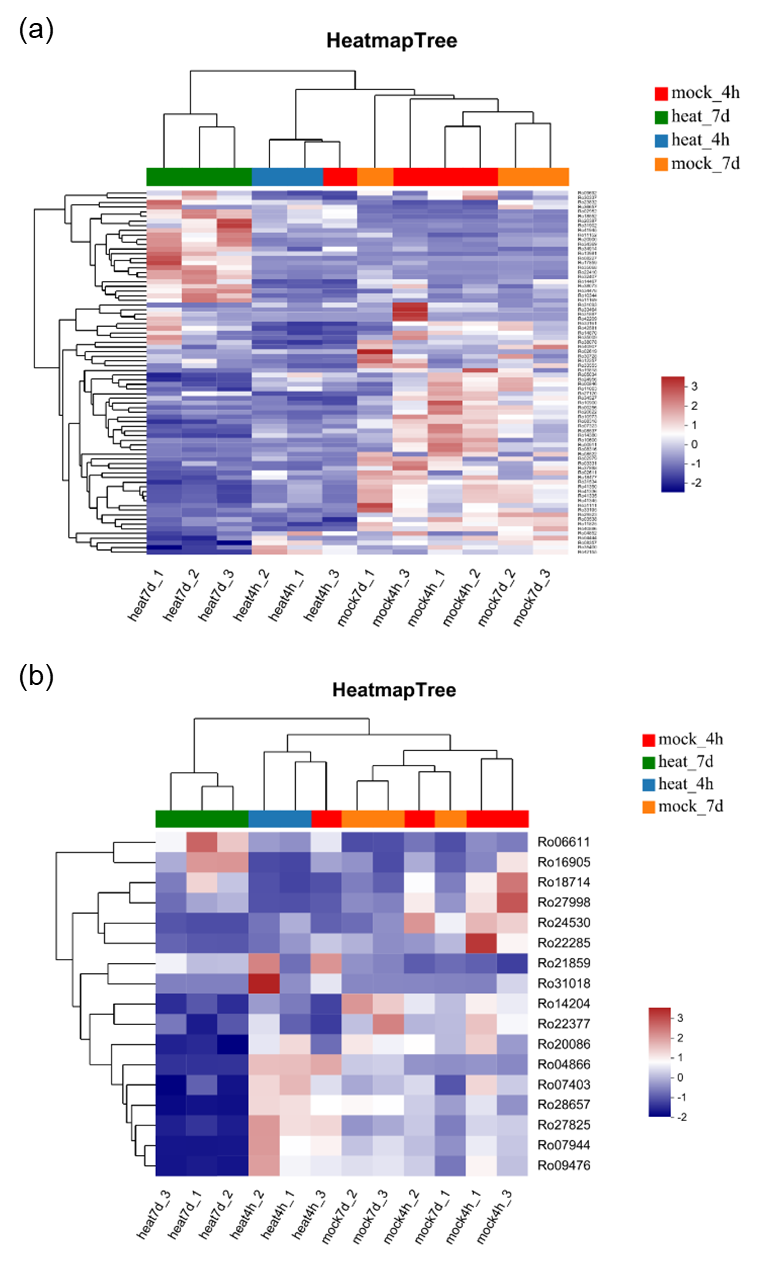


**Suppl. Fig. S7** Heatmap of sucrose and starch metabolism as well as circadian rhythm pathway-related DEGs under heat stress. **a** The heatmap of sucrose and starch metabolism pathway-related DEGs in *R. moulmainense* leaves after heat and mock treatment for 4 hours and 7 days. **b** The heatmap of circadian rhythm pathway-related DEGs in *R. moulmainense* leaves after heat and mock treatment for 4 hours and 7 days.


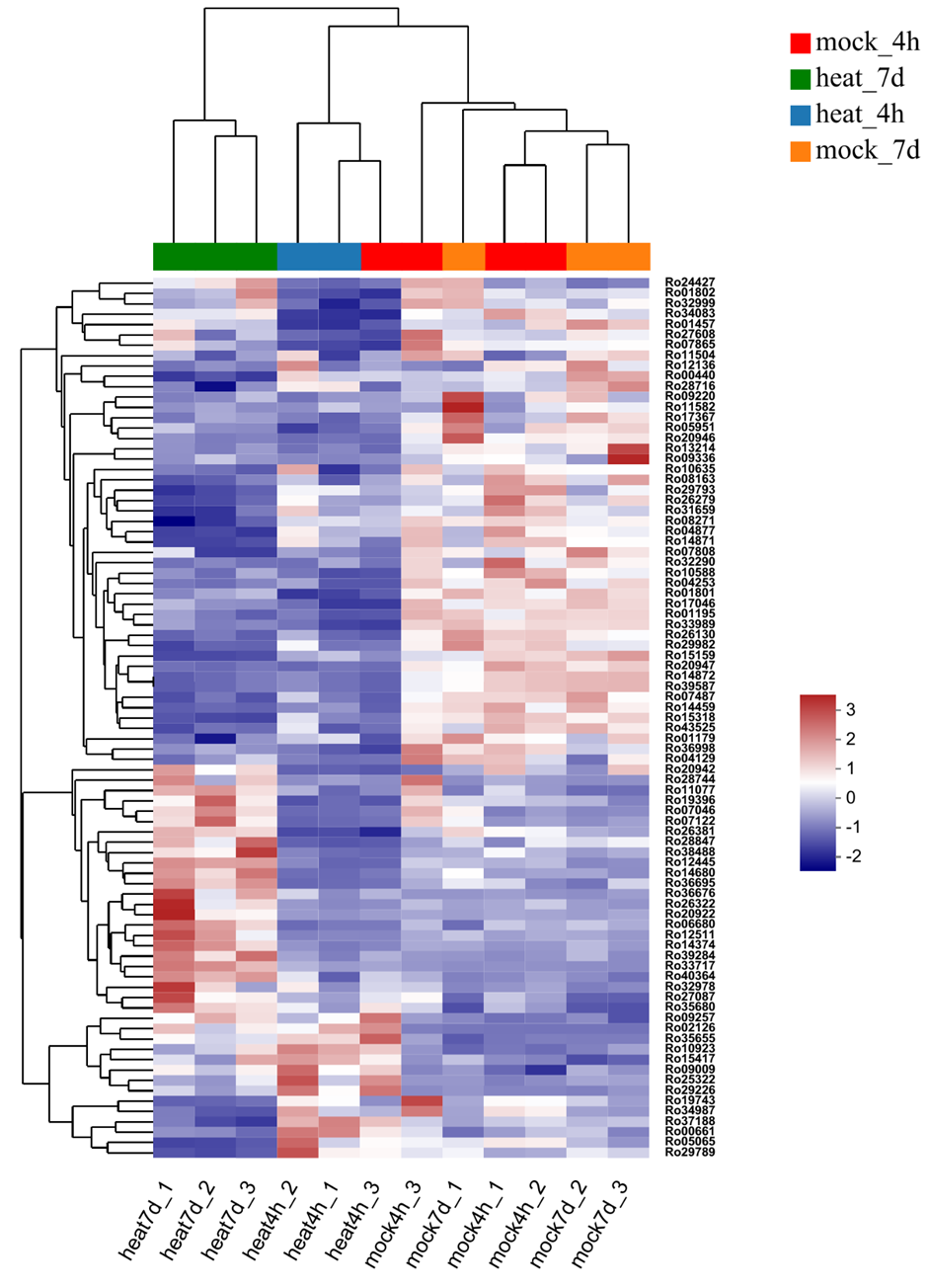


**Suppl. Fig. S8** The hormone signaling is interfered under heat stress. The heatmap of hormone signaling pathway-related DEGs in *R. moulmainense* leaves after heat and mock treatment for 4 hours and 7 days.


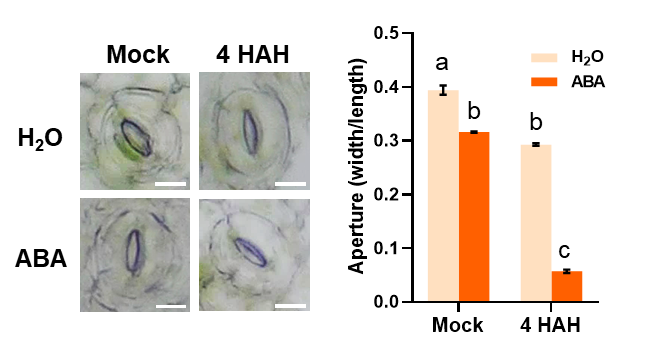


**Suppl. Fig. S9** The stomatal aperture under heat stress. Representative stomatal images and stomatal apertures in the heat- and mock-treatment *R. moulmainense* leaves under H_2_O or ABA treatment at 4 HAH. Bars = 10 μm. Mean values ± SE. The experiments were performed two times and had four biological replicates per treatment. Different letters above the bars indicate the statistically significant differences between the treatments, determined by a one-way ANOVA test followed by the Tukey’s multiple test (*P* < 0.05).


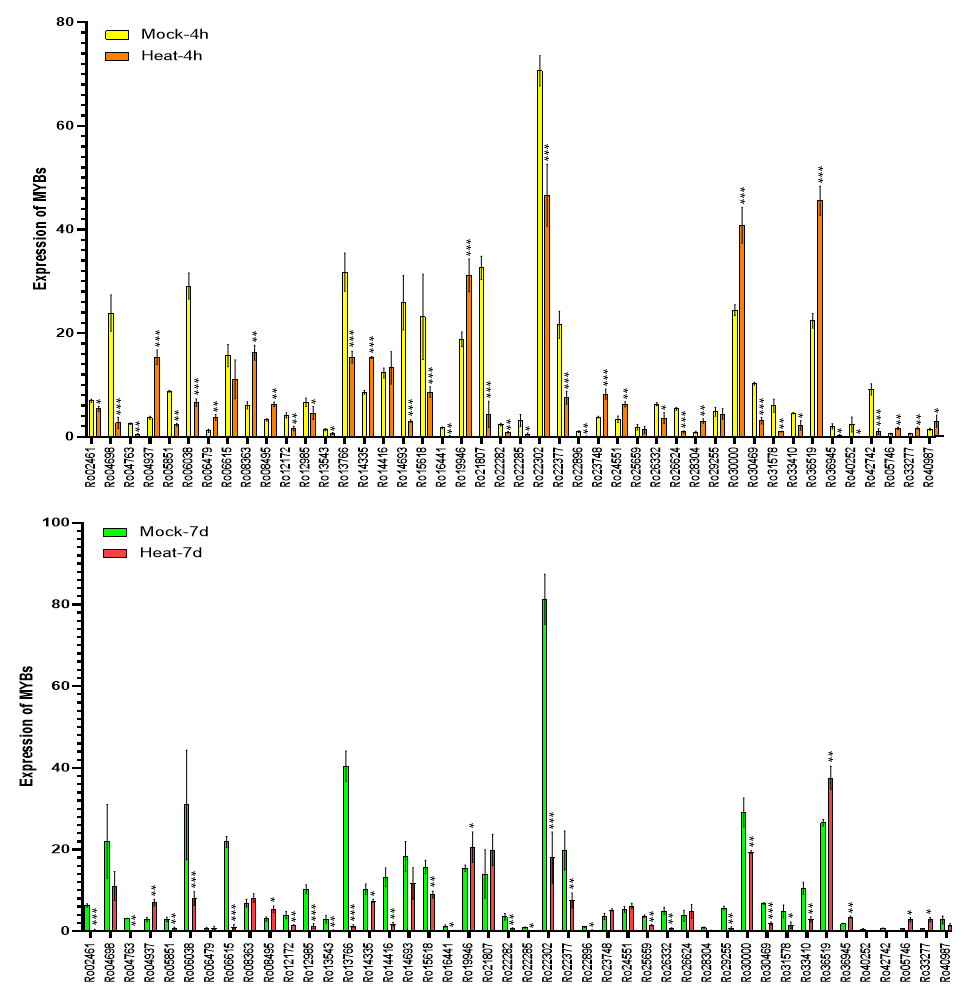


**Suppl. Fig. S10** The *MYBs* family in responding to heat stress. The expression of ABA-dependent MYBs transcript factors in *R. moulmainense* leaves after heat and mock treatment for 4 hours and 7 days. Mean values ± SE, *n* = 3. Asterisks indicate significant differences between treatments (*, *P* < 0.05, **, *P* < 0.01, ***, *P* < 0.001), determined using the two-tailed Student’s *t*-test.


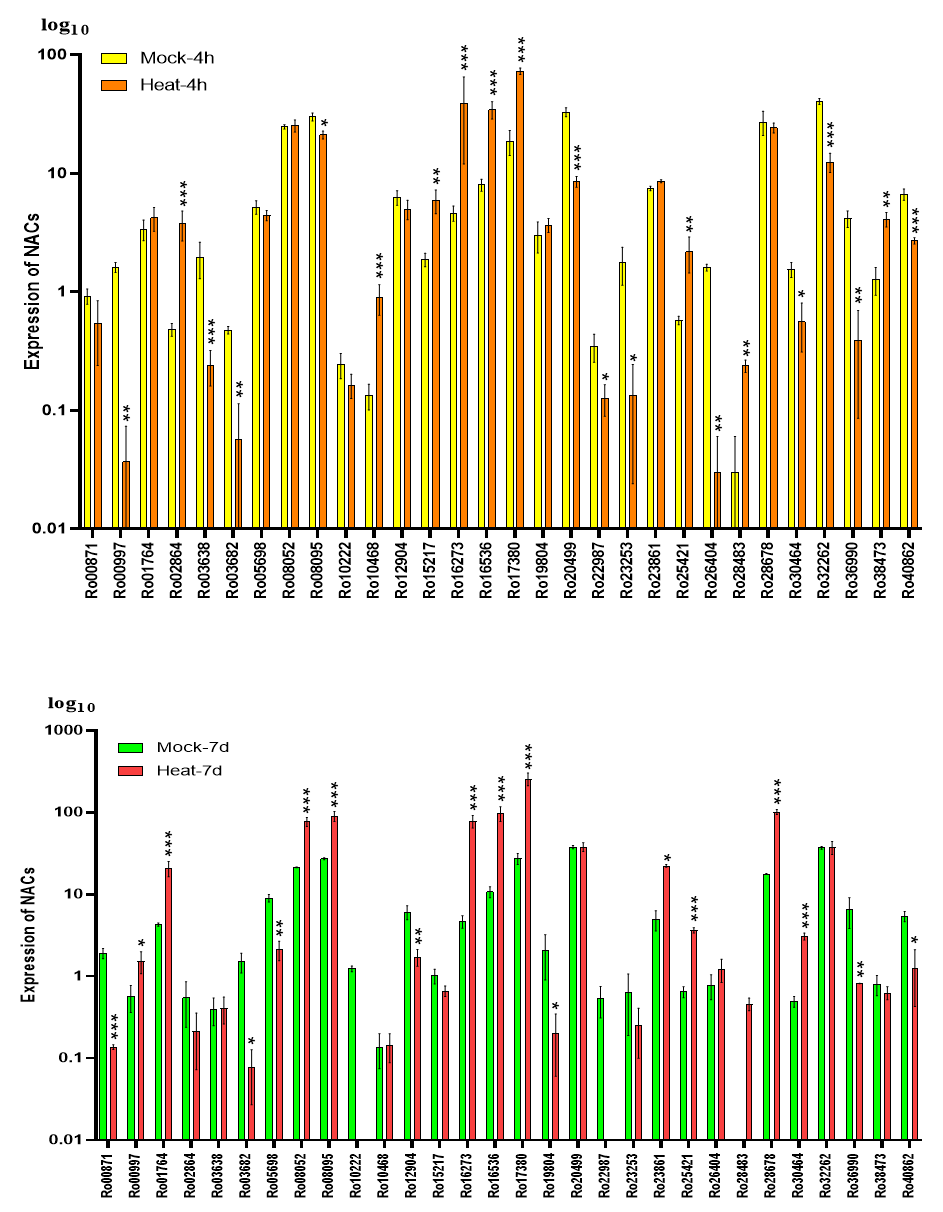


**Suppl. Fig. S11** The *NACs* family in responding to heat stress. The expression of NACs transcript factors in *R. moulmainense* leaves after heat and mock treatment for 4 hours and 7 days. Mean values ± SE, *n* = 3. Asterisks indicate significant differences between treatments (*, *P* < 0.05, **, *P* < 0.01, ***, *P* < 0.001), determined using the two-tailed Student’s *t*-test.


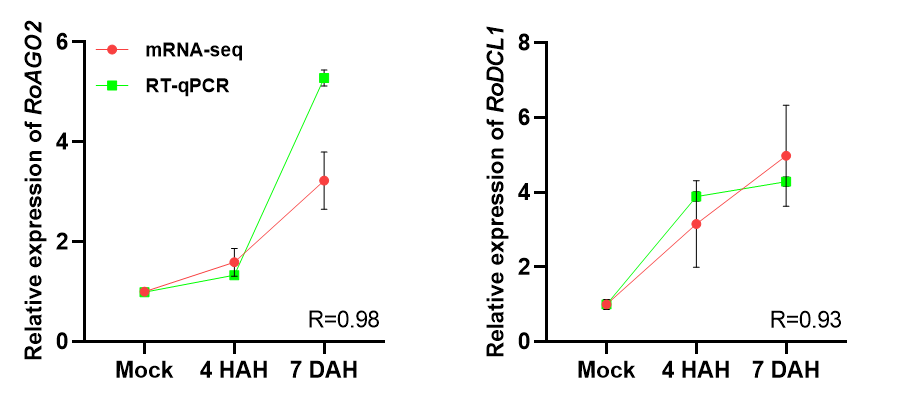


**Suppl. Fig. S12** Verfication for mRNA-seq data about small RNA regulatory pathway by RT-qPCR. The expression of *RoGAPDH* was used as the control. The expression profiles for *RoAGO2* and *RoDCL1* genes were highly consistent between mRNAseq data and RT-qPCR. *R* represents the correlation coefficient between the expression patterns of mRNA-seq data and RT-qPCR results, determined using the two-tailed Student’s *t*-test, and mean values ±SE, *n* = 3. HAH, hours after heat stress, DAH, days after heat stress.


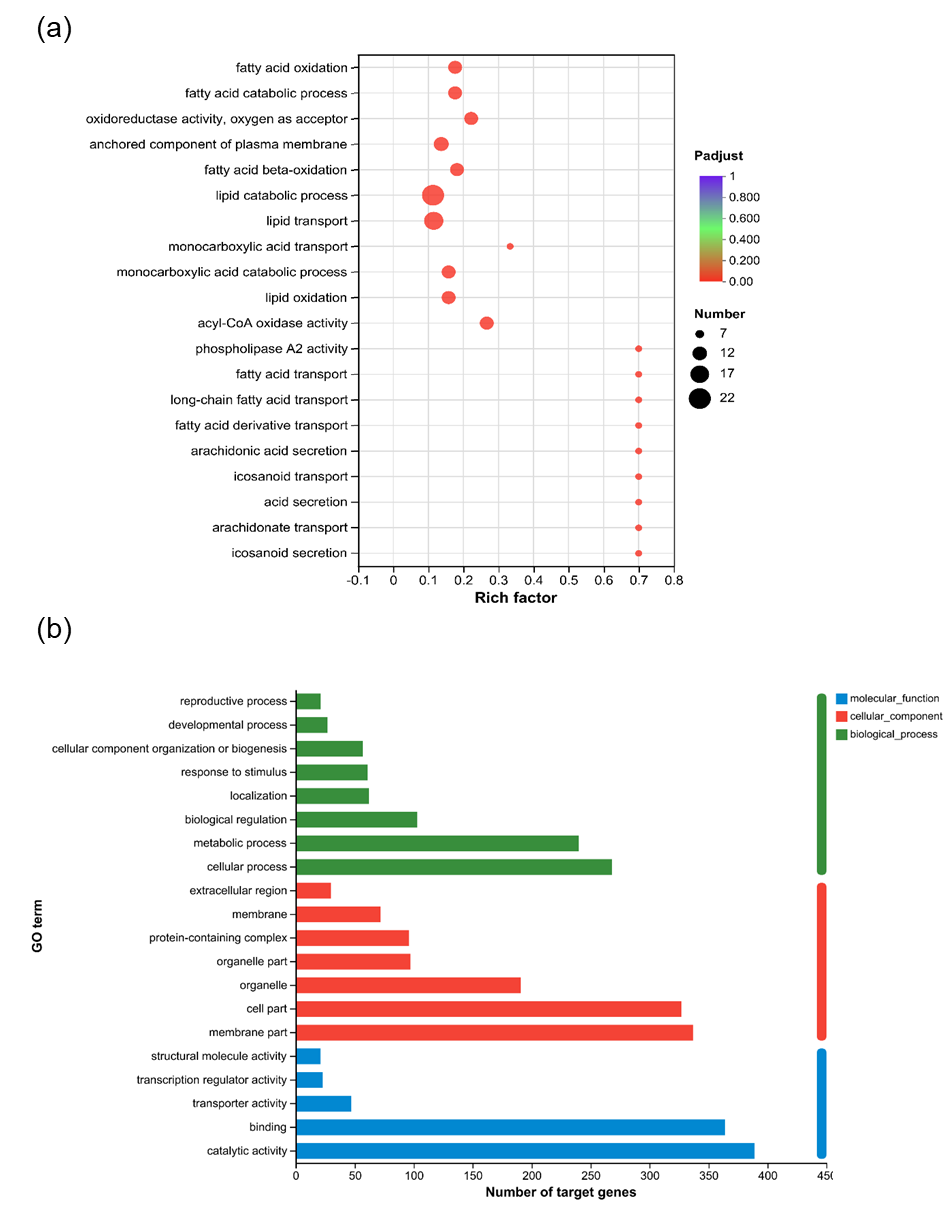


**Suppl. Fig. S13** GO enrichment and annotation analysis of different expressed miRNAs target genes. Rich Factor = the abundance or enrichment of certain transcripts in the selected transcript set compared to the entire set of transcripts in the species. Each treatment consists of 3 biological replicates.


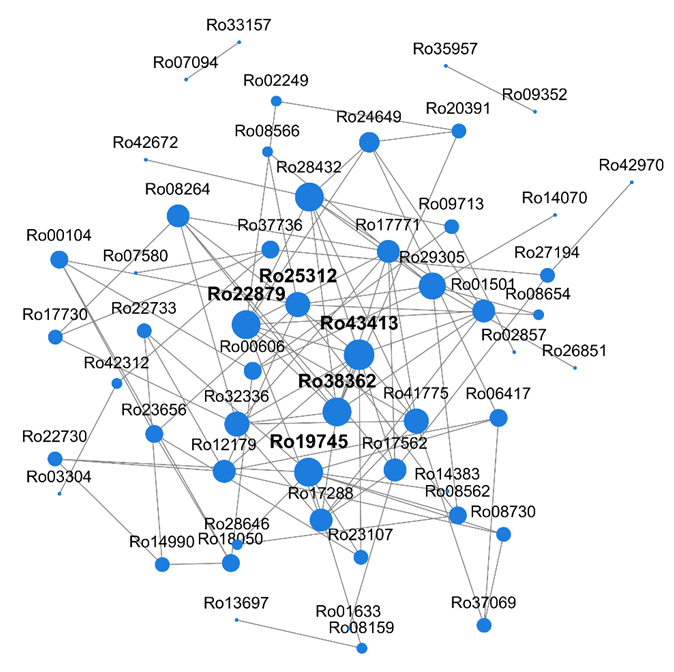


**Suppl. Fig. S14** Co-expression network of different expressed miRNAs target genes.


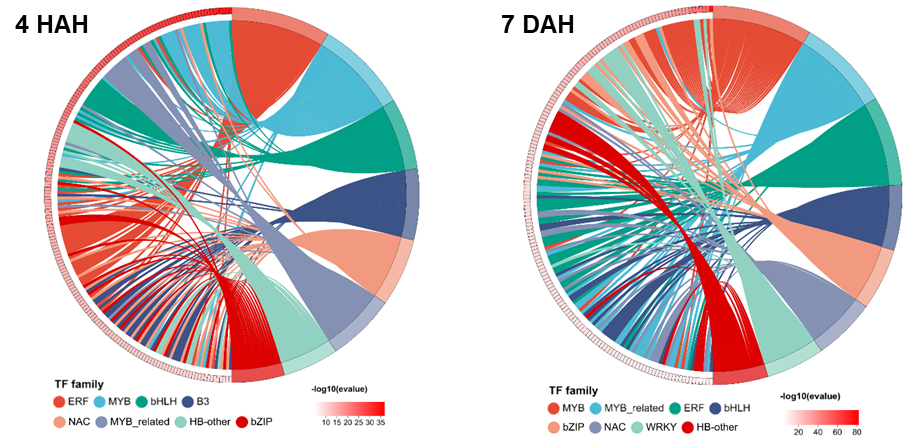


**Suppl. Fig. S15** Statistical analysis of the main differential transcription factors at 4 HAH and 7 DAH.
